# Combined SAXS/EM Based Models of the *S. elongatus* Post-Translational Circadian Oscillator and its Interactions with the Output His-Kinase SasA

**DOI:** 10.1371/journal.pone.0023697

**Published:** 2011-08-24

**Authors:** Rekha Pattanayek, Dewight R. Williams, Gian Rossi, Steven Weigand, Tetsuya Mori, Carl H. Johnson, Phoebe L. Stewart, Martin Egli

**Affiliations:** 1 Department of Biochemistry, School of Medicine, Vanderbilt University, Nashville, Tennessee, United States of America; 2 Department of Molecular Physiology and Biophysics, School of Medicine, Vanderbilt University, Nashville, Tennessee, United States of America; 3 DND-CAT Synchrotron Research Center, Northwestern University, Advanced Photon Source, Argonne National Laboratory, Argonne, Illinois, United States of America; 4 Department of Biological Sciences, Vanderbilt University, Nashville, Tennessee, United States of America; Massachusetts Institute of Technology, United States of America

## Abstract

The circadian clock in the cyanobacterium *Synechococcus elongatus* is composed of a post-translational oscillator (PTO) that can be reconstituted *in vitro* from three different proteins in the presence of ATP and a transcription-translation feedback loop (TTFL). The homo-hexameric KaiC kinase, phosphatase and ATPase alternates between hypo- and hyper-phosphorylated states over the 24-h cycle, with KaiA enhancing phosphorylation, and KaiB antagonizing KaiA and promoting KaiC subunit exchange. SasA is a His kinase that relays output signals from the PTO formed by the three Kai proteins to the TTFL. Although the crystal structures for all three Kai proteins are known, atomic resolution structures of Kai and Kai/SasA protein complexes have remained elusive. Here, we present models of the KaiAC and KaiBC complexes derived from solution small angle X-ray scattering (SAXS), which are consistent with previous EM based models. We also present a combined SAXS/EM model of the KaiC/SasA complex, which has two N-terminal SasA sensory domains occupying positions on the C-terminal KaiC ring reminiscent of the orientations adopted by KaiB dimers. Using EM we demonstrate that KaiB and SasA compete for similar binding sites on KaiC. We also propose an EM based model of the ternary KaiABC complex that is consistent with the sequestering of KaiA by KaiB on KaiC during the PTO dephosphorylation phase. This work provides the first 3D-catalogue of protein-protein interactions in the KaiABC PTO and the output pathway mediated by SasA.

## Introduction

**Figure 1:**
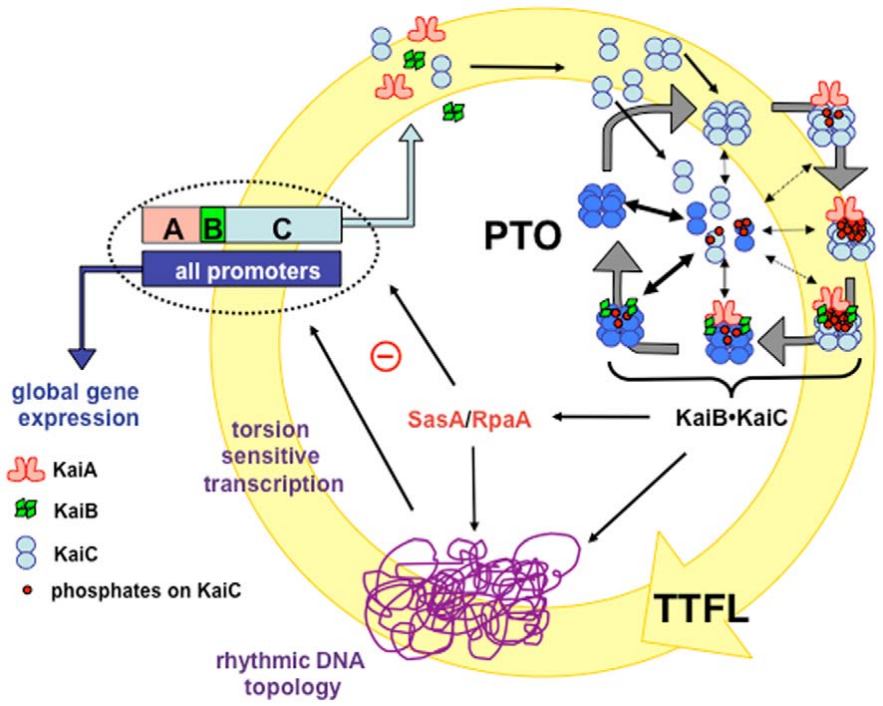
In the *S. elongatus* circadian clock the post-translational oscillator (PTO) is embedded in a transcription-translation feed loop (TTFL). Key features of the oscillator are: (i) Mediation of global gene expression by rhythmic modulation of promoters including those driving the cluster of core clock protein genes, *kaiA*, *kaiB* and *kaiC*; (ii) modulation of global promoter activity by rhythmic DNA torsion and/or transcription factor activity (i.e. RpaA, signaled by the PTO output His kinase SasA); (iii) regulation of DNA topology and transcription factors by rhythmic phosphorylation and dephosphorylation of the KaiC homo-hexameric protein; (iv) robustness conferred by synchronization of KaiC hexamer status through monomer exchange in the PTO; (v) modulation of amplitude or phase setting by newly synthesized non-phosphorylated KaiC hexamers or monomers feeding into pre-existing hexamers; and (vi) a core PTO composed of KaiA, KaiB, KaiC and ATP, whereby KaiC has kinase, phosphatase (putative conformational changes between the two states are indicated by dark- and light-blue coloring of hexamers), and ATPase activities, KaiA enhances phosphorylation and KaiB antagonizes KaiA.

The circadian clock in the cyanobacterium *Synechococcus elongatus* (*S. elongatus*) ticks in the absence of transcription and translation [1]. A mixture of the KaiA, KaiB and KaiC proteins in the presence of ATP and Mg^2+^ exhibits stable oscillations *in vitro* between the hypo- and hyper-phosphorylated states of KaiC with a ∼24-h period [2]. Not only does the period of this post-translational oscillator (PTO) match that exhibited by the clock *in vivo* under light/dark conditions, but the PTO is temperature-compensated, mutagenesis of its constituent proteins *in vitro* triggers alterations of the period that are similar to those observed with mutant strains *in vivo* [2], and it is able to undergo phase changes as demonstrated by temperature jumps [3]. Other rhythms that prevail in *S. elongatus* include promoter activity and a daily compaction and expansion of the chromosome that may play a role in clock-controlled gene expression [4] (**Figure 1**). Interestingly, the timing of cell division that occurs every 10.5 h is controlled by the circadian oscillator, but the behavior of the latter is unaffected by division, as indicated by identical phases of the clocks in parent and offspring cells [5]. Similarly, various levels of KaiC expression as a result of exposure of *S. elongatus* cells to different light/dark cycles leave the PTO invariant, thus providing evidence that it represents the master timer and that the transcription-translation feedback loop (TTFL) is under the control of the PTO [6] (**Figure 1**).

The protein-protein interactions underlying the PTO are highly dynamic. Rather than particles composed of Kai proteins that move in lockstep, the concentrations of the free KaiA, KaiB and the phosphorylated and non-phosphorylated KaiC proteins as well as those of their binary and ternary complexes oscillate over the daily cycle [3], [5], [7]. KaiC is a homo-hexamer [8]–[10] that possesses auto-kinase, auto-phosphatase and ATPase activities [11]–[14]. At the beginning of the 24-h period, rapid and repeated action of the KaiA dimer on the KaiC hexamer increases KaiC's phosphorylation [7]. KaiB is a KaiA antagonist [11], [12], [15]–[17] and interacts with KaiC in the hyperphosphorylated state [3], [5], [7]. Apart from dephosphorylation, KaiB binding induces KaiC subunit exchange, a process that is crucial for maintaining a high-amplitude oscillation [3], [7]. Moreover, KaiB's role as an antagonist to KaiA may involve sequestration of the latter in ternary KaiABC complexes in which KaiA is unable to act as an enhancer of KaiC phosphorylation [18], [19].

Three-dimensional structures for KaiA [16], [20], [21]–[23], KaiB [20], [24]–[26], and KaiC [10] proteins or individual domains from a variety of cyanobacterial strains have been available for some time (reviewed in [27]). KaiC, a Ser-Thr kinase, contains two phosphorylation sites in the C-terminal (KaiCII) half, and harbors ATPase activity in the N-terminal (KaiCI) half [28]–[30]. Phosphorylation of the S431 and T432 side chains in KaiCII follows a strict order such that a phosphate is first transferred to T432 and also first removed from T432; with the phosphorylation order represented by: TS→pTS→pTpS→TpS→TS [31], [32]. Recent analyses of the role played by the T426 residue that engages in a H-bonding interaction with phosphorylated S431 indicate that this threonine needs to be phosphorylatable for the clock to function properly [33]. Indeed, phosphorylation at T426 was observed in the crystal structures of the KaiC S431A single and T432E/S431A double mutant proteins [30]. The priority of T432 in terms of phosphorylation is likely a consequence of this residue being consistently positioned closer to the γ-phosphate of ATP compared to S431 in crystal structures of KaiC. Similarly, the persistence of the KaiC TpS band as tracked by gels during the dephosphorylation phase of the clock, when KaiB is bound to KaiC, can be rationalized by the more sheltered environment of S431 and the interactions of its phosphate group with nearby residues T426 and H429 [30].

How KaiA and KaiB exert their opposite influences over the KaiC phosphorylation status constitutes a central problem in the analysis of the KaiABC clock mechanism. While atomic-resolution structures of binary complexes have remained elusive, we have used a hybrid structural approach to characterize the interactions of KaiA and KaiB with KaiC. Combining X-ray crystallographic [10], [23], solution NMR [22] and electron microscopy (EM) data, we have modeled so-called ‘tethered’ and ‘engaged’ configurations of the KaiA-KaiC (KaiAC) complex [34]. In our KaiAC models a flexible linker between the KaiA dimer and the KaiC hexamer is formed by KaiC residues that are near the C-terminus and form an S-shaped loop (aa 485–497) in the KaiC crystal structure. In both the ‘tethered’ and the ‘engaged’ configurations this S-shaped loop has to unravel and this appears to increase the flexibility of the six KaiCII domains with respect to each other. As phosphorylation of the KaiC S431 and T432 residues occurs across subunit interfaces, increased flexibility would tend to enhance auto-kinase activity [4], [5], [27], [35]. This is consistent with the observation that the S-shaped loop is important for locking KaiC in either the hypo- or the hyper-phosphorylated state [36] and the earlier observation that a single KaiA dimer is able to drive KaiC to the hyper-phosphorylated state [37]. With regard to the KaiB-KaiC (KaiBC) complex, we have established that KaiB binds to the C-terminal KaiCII ring [26]. In our EM based KaiBC model, two KaiB dimers occupy opposite rims of the dome-shaped surface of KaiCII, thereby preventing the ‘engaged’ configuration of the KaiA dimer and blocking further KaiA action on KaiC. Our EM-based model differs from a model proposed by others using small angle X-ray scattering (SAXS) that had a KaiB tetramer bound to the KaiC hexamer [38]. Another notable difference between these two KaiBC models concerns the central channel of the KaiC hexamer. In our EM based model KaiB does not obscure the channel, whereas in the Akiyama *et al.* SAXS model the KaiB tetramer blocks the channel.

To further analyze the protein-protein interactions driving the *S. elongatus* circadian PTO, we used SAXS in combination with EM and X-ray crystallography to establish and confirm models of the binary KaiAC and KaiBC complexes, the complex between KaiC and the output His kinase SasA, and the ternary KaiABC complex. Unlike KaiA and KaiB, which are active during either the phosphorylation or dephosphorylation phase of the clock cycle, SasA initially appeared to be constitutively bound to KaiC (immunoprecipitations based on two time points [39]). However, subsequent studies established that SasA interacts with KaiC in a circadian fashion, binding more abundantly during the subjective night [40]. Together with the transcription factor RpaA, SasA forms a two-component regulatory system that mediates between the phosphorylation state of the PTO and global transcription rhythms [41] (**Figure 1**). It is known that the N-terminal sensory domain of SasA (N-SasA) contacts KaiC directly and shares considerable sequence similarity with KaiB. However, the NMR solution structure of N-SasA disclosed some important differences to KaiB. Prior to our current study no details had emerged on the binding mode of SasA to KaiC or on whether or not KaiB and SasA compete for the same binding site on KaiC [42]. To enhance the binding interactions in the complexes we made use of mutant KaiC proteins such as the T432A/S431A double mutant (KaiC-aa; with enhanced affinity to KaiA) and the T432E/S431E double and S431D single mutants (KaiC-ee and KaiC-dT; with enhanced affinity to KaiB). We have determined the crystal structure of the KaiC-ee hexamer and identified specific changes at its active site relative to the wild-type KaiC structure in its hyper-phosphorylated state [10] that are indicative of conformational plasticity at the subunit interface.

Overall, our work establishes the basic interaction motifs between the Kai proteins in binary and ternary complexes. EM data is presented indicating that SasA and KaiB compete for the same binding site on the KaiCII side of KaiC. A combination of EM and SAXS data is used to model the basic interaction mode between SasA and KaiC. In addition, a structural model is presented for the sequestration of KaiA during the cycling reaction, which has been linked biochemically to the proper functioning of the PTO.

## Results

### SAXS Models of Individual *S. elongatus* Kai Proteins

**Figure 2:**
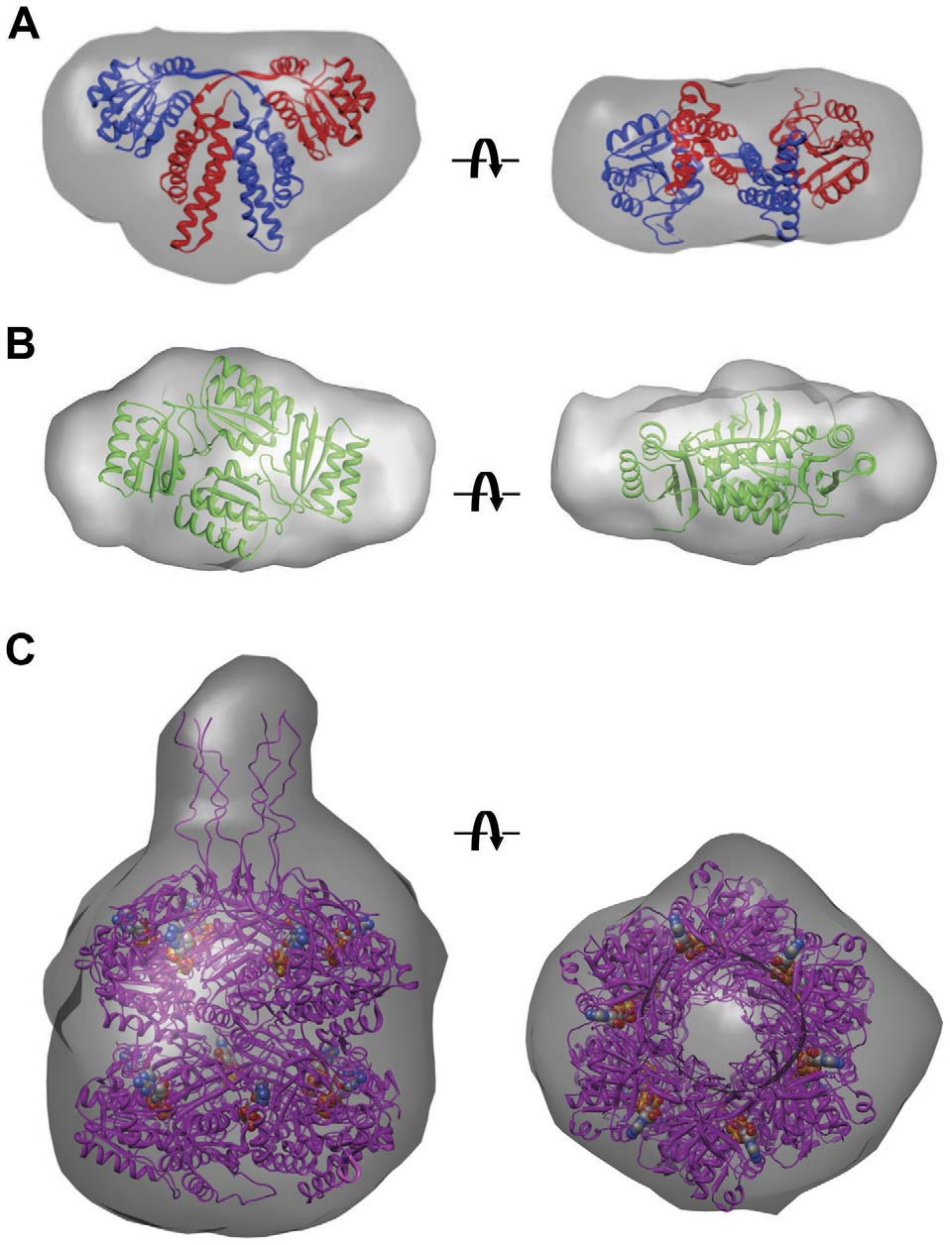
SAXS envelopes for individual Kai proteins from *S. elongatus*. (A) Envelope for KaiA with the crystal structure of the dimer (PDB ID 1R8J [23]; http://www.rcsb.org) modeled into it. The views are approximately perpendicular to the molecular dyad (left) and along it (right). Subunits of the domain-swapped dimer are colored in red and blue. (B) Envelope for KaiB with the crystal structure of the tetramer from *Thermosynechococcus elongatus* (PDB ID 2QKE [26]) modeled into it. The views are approximately along the dyad relating dimers (left) and perpendicular to it (right). (C) Envelope for KaiC hexamer (KaiC-aa mutant) with the crystal structure of wt-KaiC (PDB ID 3DVL [30]) modeled into it. The views are approximately perpendicular to the molecular sixfold rotation axis (left) and along it (right). The conformations of C-terminal tails depicted in the model are based on the one fully traced tail of subunit A in the crystal structure of wild type KaiC from *S. elongatus* refined to 2.85 Å [34]. Only two of the chains could be completely traced up to the C-terminal residue S519, and the conformations of individual tails are affected by the packing of hexamers in the crystal. ATP molecules are shown in space filling mode. The orientations of the crystallographic models inside the individual SAXS envelopes were optimized by rigid body refinement. The symbols indicate rotations of 90 degrees.

To establish a baseline for interpretation of SAXS envelopes of Kai protein complexes, we first examined individual Kai proteins. The three proteins from *S. elongatus* were expressed in *E. coli* either as GST-fusion proteins (KaiA, KaiB and KaiC; GST tags were cleaved off for further work) or with a C-terminal (His)_6_ tag as previously described [3], [10]. SAXS data were collected using protein solutions in the concentration range of between 0.5 and 2.0 mg/mL on the setup of the DND-CAT synchrotron research center at sector 5 of the Advanced Photon Source (APS, Argonne IL) [43]. The three proteins were well behaved and the solutions were virtually free of aggregation as evidenced by linear Guinier plots (see **Figures S1, S2, S3**). Selected parameters such as the experimental and theoretical *R*_G_ values are listed in **Table 1**. SAXS molecular envelopes were calculated with the programs DAMMIN and GASBOR [44]; in each case the solution structures were initially generated using P1 symmetry. Crystal structures were docked into SAXS envelopes manually and the initial orientations subsequently optimized by rigid-body refinement using the program CHIMERA [45]. The close correspondence between the SAXS envelopes and crystallographic models (**Figure 2**) is borne out by similar values of the experimental *R*_G_ and those calculated from crystallographic coordinates (**Table 1**). The SAXS results on the individual Kai proteins confirm their quaternary states in solution (KaiA, dimer; KaiB, tetramer; and KaiC, hexamer).

**Table 1:** Overview of SAXS data for Kai proteins and their binary complexes.

Protein/Complex	*R*_G_ [P(r)] [Å]	*R*_G_ (Guinier)[Å]	Model	*R*_G_ (Model) [Å]	NSD1)
**KaiA**	32.42±0.03	33.3±0.2	PDB ID 1R8J [23]	34.0	0.95 (0.03)
**KaiB**	33.66±0.01	30.3±0.1	PDB ID 2QKE [26]	30.3	0.84 (0.02)
**KaiC**	46.36±0.02	47.4±0.1	PDB ID 3DVL [30]	42.0	0.66 (0.05)
**KaiAC**	55.80±0.07	59.7±0.4	KaiC with tethered KaiA [34]	66.1	0.76 (0.03)
**KaiBC**	46.90±0.06	46.2±0.2	KaiBC EM model [26]	45.4	0.89 (0.01)
**KaiC-SasA**	49.70±0.10	47.7±0.5	KaiC-SasA model (**Fig. S9**)	52.6	0.54 (0.02)

1) Normalized spatial discrepancy (standard deviation in parentheses).

The KaiA protein adopts a domain-swapped dimer in the crystal [23] and the SAXS envelope neatly surrounds the coordinates of the dimer (**Figure 2A**). KaiA has an N-terminal, bacterial receiver-like domain and a C-terminal four-helix bundle that serves to sustain the dimer. KaiB adopts a thioredoxin-like fold (but lacks the catalytic cysteines of the thioredoxines) and consistently forms a dimer of dimers in crystal structures [20], [24]–[26]. The KaiB SAXS envelope matches the shape of a crystallographic tetramer, although some extra room remains in the SAXS envelope, which we postulate is due to the flexible N- and C-terminal peptide tails which are not fully resolved in the crystal structures (see refs. [24], [26]) (**Figure 2B**). The formation of a KaiB tetramer as established by SAXS is consistent with earlier light scattering data [24], and confirms that the tetramer is the preferred oligomeric state of KaiB in solution. The SAXS envelope for KaiC is consistent with the shape of the homo-hexamer seen in the crystal structure [10]. KaiC resembles a double doughnut comprised of N-terminal CI and C-terminal CII rings, with a height and diameter of ca. 100 Å and a subtle restriction at the waist. The SAXS-based molecular envelope shows a narrow protrusion on one side (**Figure 2C**), which we postulate is generated by the C-terminal peptide tails that emerge from the dome-shaped surface of the KaiCII ring [34]. No such tails emerge from the opposite side, the KaiCI side, of the hexamer. Interestingly, EM reconstructions based on negative stain EM or cryoEM data at resolutions of around 20 Å lack this bulge [8], [9], [34]. The flexible C-terminal tails of KaiC are difficult to resolve by negative stain EM. However after adding KaiA, which binds to the KaiC C-terminal tails, we observed a clear difference between class sum images of KaiAC formed with wild-type KaiC and class sum images of KaiAC formed with C-terminally truncated KaiC [34]. The observation of a bulge in the KaiC SAXS envelope for the tails suggests that one may consider SAXS envelopes to correspond to lower resolution representations of proteins with amplified representation of flexible regions compared to moderate resolution EM structures. Observation of the C-terminal peptide bulge in the SAXS envelope provides the benefit of enabling us to distinguish the CI and CII ends of the KaiC hexamer.

### SAXS Model of the KaiAC Complex

**Figure 3:**
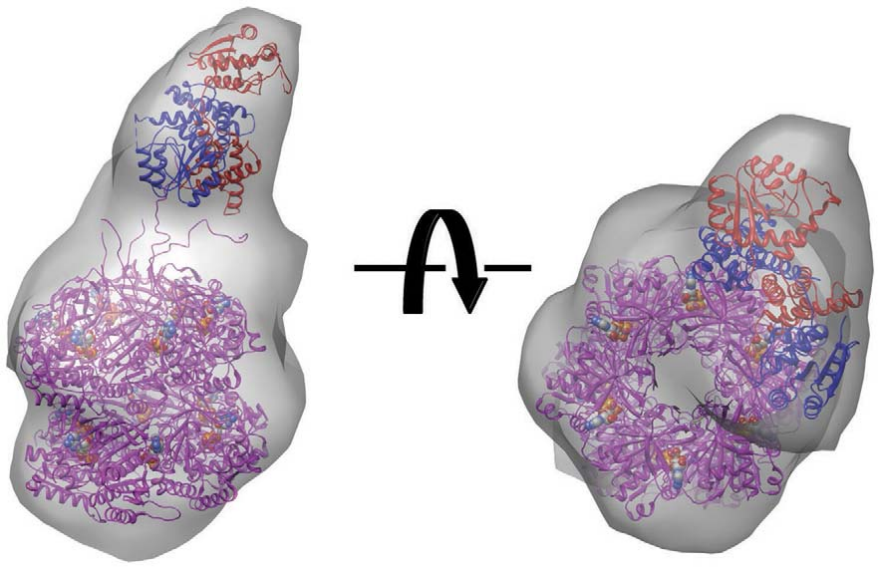
KaiAC complex by SAXS. The complex was formed with *S. elongatus* KaiA and the KaiC-aa mutant. The shape of the protrusion above the KaiC barrel (magenta) is indicative of a single KaiA dimer (subunits colored in red and blue), bound to a KaiCII C-terminal peptide. The position of the KaiA dimer at some distance from the KaiCII surface is reminiscent of the ‘tethered’ model of the complex determined by EM [34]. The symbol indicates a rotation of 90 degrees.

KaiA stimulates KaiC phosphorylation and the KaiCII domains of KaiC harbor the kinase and phosphatase activities. Mutations of key KaiC residues, including the phosphorylation residues T432 and S431, cause subtle differences in the active-site geometry, affect the clock period, or abolish rhythmicity [28], [30], [33]. Although KaiA is able to bind both hypo- and hyper-phosphorylated forms of KaiC [7], we reasoned that the KaiAC interaction might be enhanced with mutant forms of KaiC. Various single and double mutants of KaiC were evaluated. The KaiC T432A/S431A double mutant (KaiC-aa) seems to enhance the formation of a stable KaiAC complex. We relied on native polyacrylamide gel electrophoresis (PAGE) to assay binding between KaiA and KaiC [34], establish optimal conditions for formation of the complex, and minimize the presence of the free proteins in the SAXS samples. Representative scattering curves and the corresponding Guinier plots for the KaiAC complex are depicted in **Figure S4**. The calculated envelope displays a protrusion at one end of the KaiC barrel that is consistent with binding of a single KaiA dimer (**Figure 3**). The protrusion is assigned to the KaiCII end because an NMR structure showed conclusively that the KaiA dimer binds to the C-terminal peptide tail of KaiC [22]. The earlier KaiAC EM reconstruction supported the idea of multiple orientations of the KaiA dimer relative to the KaiC hexamer [34]. The KaiAC SAXS envelope more closely resembles the so-called ‘tethered’ EM model of the complex between KaiA and KaiC. The SAXS envelope represents a time averaged form of the complex, and therefore we deduce that the predominant position for KaiA in the KaiAC complex resembles the ‘tethered’ form with KaiA ∼35 Å from the KaiC hexameric barrel. Both the EM structure and SAXS envelope of KaiAC are consistent with a somewhat flexible linker between the two proteins and a predominant stoichiometry with one dimer of KaiA bound to one hexamer of KaiC.

### Crystal Structure of the KaiC T432E/S431E Mutant and Conformational Plasticity of KaiC

**Figure 4:**
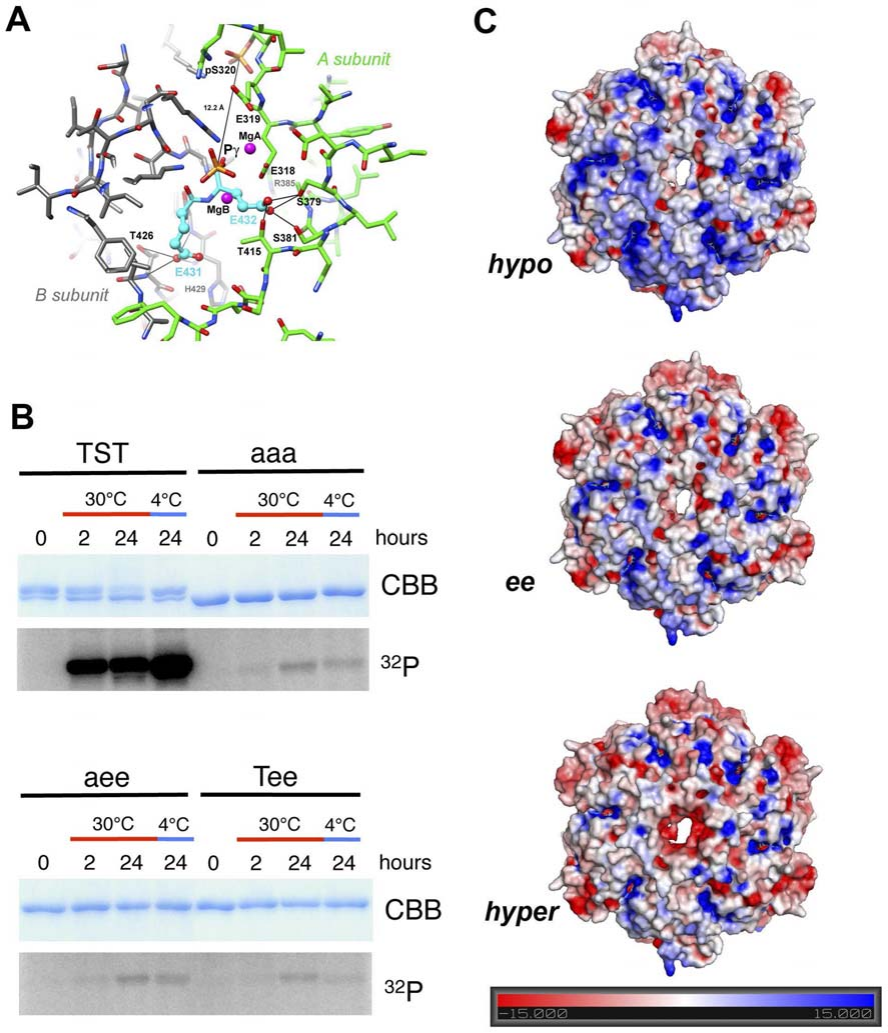
Crystal structure of the *S. elongatus* KaiC-ee mutant and KaiC conformational plasticity. (A) The crystal structure of KaiC-ee reveals phosphorylation at S320 in the A and F subunits. The figure depicts a portion of the interface between the A (carbons green) and B (carbons gray) subunits and intra-subunit phosphorylation at S320[A] (top). Carbon atoms of E432[B] and E431[B] are highlighted in cyan (center) and hydrogen bonding interactions between E432[B] and S379[A], S381[A] and T415[A] (on the right) and between E431[B] and T426[B] (bottom) are shown as thin solid lines. The gamma phosphate (Pγ) of the ATP molecule bound between the A and B subunits is shown, as well as the distance (12.7 Å) between it and the phosphate of S320. (B) *In vitro* phosphorylation patterns of wt-KaiC (T426/S431/T432 = TST), and the KaiC-aaa (T426A/S431A/T432A), KaiC-aee (T426A/S431E/T432E) and KaiC-ee (T426/S431E/T432E) mutants. All mutants exhibit phosphorylation in the ^32^P assay (albeit at a much lower level than the wt protein), consistent with a new phosphorylation site outside the known triad T432, S431 and T426. (C) Calculated electrostatic surface potentials for KaiC (hypo-phosphorylated), KaiC-ee and P-KaiC (hyper-phosphorylated) (from top to bottom) with the hexamers viewed from their C-terminal ends. Markedly different polarizations might well contribute to KaiB's ability to distinguish between the hypo- and hyper-phosphorylated states and preferentially bind the hyper-phosphorylated state.

Various mutants, including the KaiC T432E/S431E double mutant (KaiC-ee) have been designed to mimic the hyper-phosphorylated state of KaiC [6], [46]. Glutamate or asparate substitution of phosphorylation sites is a common approach to emulate the phosphorylated state of a protein. Glu, in particular, is spatially similar to both phosphorylated Thr and Ser, however Glu and Asp only contribute a single negative charge instead of the two negative charges of a phosphate group. To gain insight into the active site properties of the KaiC-ee mutant we determined its crystal structure at 3.0 Å resolution. A summary of data collection and refinement parameters is given in **Table S1** and an example of the quality of the final electron density is depicted in **Figure S5**. Analysis of the region around the two introduced glutamates reveals several important changes compared to wt-KaiC. First, in the wt-KaiC structure both S431 and T432 are engaged in close contact with R393 from the same subunit [4], [30], but neither E431 nor E432 are close to R393 in the KaiC-ee structure. Secondly, instead of phosphorylated T432 residues stitching together adjacent subunits by forming a salt bridge to R385 as observed in the crystal structure of wt-KaiC [28], the side chains of E432 residues have shifted away from this arginine (**Figure 4A**). Notably the distances between all pairs of side chain oxygen and nitrogen atoms from E432 and R385, respectively, exceed 5 Å in the six subunits of KaiC-ee. Instead E432 side chains lean over toward three serine and threonine residues, S379, S381 and T415, in the adjacent subunit. In all subunits at least three hydrogen bonds are established between the carboxylate moiety of E432 and the Oγ oxygen atoms of these three amino acids. It is unclear whether these newly formed interactions provide more stability than the salt bridges across subunits in the wt-structure. What is clear, however, is that E432 does not completely mimic pT432. In the wt-KaiC structure distances between phosphate oxygens of phosphorylated T432 residues and side chain oxygens of S379, S381 and T415 residues exceed 4 Å in all six subunits. In contrast, the side chain of the adjacent residue E431 does not fundamentally alter its orientation relative to pS431 in the wt-structure. Carboxylate moieties of E431 side chains engage in hydrogen bonds to the main chain nitrogen and/or the side chain Oγ of T426 from the same subunit.

Surprisingly, inspection of the electron density maps of the KaiC-ee mutant revealed that residues S320 in the A and F subunits carry a phosphate group (**Figures 4A** **, S5A**). If we envision the key components of the KaiC kinase active site, (ATP)Pγ, (T432)Cα and (S431)Cα, as located near the center of a sphere with a radius of ca. 12 Å, then S320 and T426 would occupy approximately the North and South poles, respectively (**Figure 4A**). In wt-KaiC it is important to note that the phosphorylation sites T432, S431 and T426 are all phosphorylated by active site residues on the adjacent subunit. In KaiC-ee S320 is phosphorylated by active site residues on the same subunit. In other words, phosphorylation of S320 does not proceed across the subunit interface but constitutes an intra-subunit process. The distance between Oγ of S320 and Pγ is closer in the A and F subunits of KaiC-ee than the average distance between Oγ of T426 and Pγ in the wt-structure (**Table 2**). This slight rearrangement in the KaiC-ee structure favors phosphorylation of the alternate S320 site. SDS-PAGE analysis of the KaiC-ee double-mutant and T426A/SA431E/T432E triple-mutant (KaiC-aee) proteins incubated with γ-^32^P-ATP shows weak residual phosphorylation that cannot occur at any of the three normal phosphorylation sites (**Figure 4B**). Clearly replacement of T426 by Ala and both S431 and T432 by Glu does not completely block phosphorylation at alternative sites. In fact, the gel assay also provides clear evidence that the triple alanine mutant of KaiC (T426A/S431A/T432A = KaiC-aaa) also exhibits phosphorylation. These results indicate that KaiC is a more promiscuous kinase than hitherto assumed. While gel-based assays can detect phosphorylation, crystallographic analyses or other high resolution biophysical studies are necessary to reveal the identity of alternative phosphorylation sites.

**Table 2:** Distance1) relationships in the crystal structure of KaiC-ee.

Subunit	d(E432)	d(431)	d(S320)
A	6.75	10.03	**13.25** (**12.76**; P)2)
B	6.60	9.97	12.32
C	7.06	9.76	12.84
D	6.65	9.50	13.19
E	6.82	9.74	12.76
F	6.98	9.75	**13.14** (**12.14**; P)2)
Average	6.81	9.79	12.923)

1) d in Å between Pγ (ATP) and Cδ of E432 or E431 or Oγ of S320.

2) S320 in subunits A and F exhibits phosphorylation.

3) For comparison, the avg. distance to T426 Oγ is 12.5 Å.

The KaiC-ee mutant was designed to mimic a permanently phosphorylated form of KaiC that is unable to oscillate between the hypo- and hyper-phosphorylated states. However, the conformational changes observed in the structure of KaiC-ee and the presence of an additional phosphorylation site have to be taken into account when interpreting functional observations of this mutant. While initial reports suggested that constitutive phosphorylation of KaiC allowed rhythmicity to proceed *in vivo* [46], it was subsequently found that cells expressing KaiC-ee exhibited a long period that was severely damped. Also these rhythms were not compensated for by changes in metabolic activity [6]. In fact, the period measured using luminescence assays *in vivo* (60 h) exceeds the published *in vitro* number (47 h) [46]. Therefore, the latest results indicate that the KaiC-ee mutant has a severely distorted *in vivo* rhythm [6] (**Figure S6**). *In vivo* assays of the mimic of the phosphorylated form at the third site, T426E, show complete loss of rhythm. This further supports the idea that the phosphorylation state of KaiC is in a delicate balance that can easily be tipped in one direction or another.

Despite the noted structural variations, the KaiC-ee mutant is clearly of use in biophysical studies and the changes in the overall structures between wt-KaiC and various phosphorylation mutants observed in the solid state (this work and [30]) and in solution [47] are not dramatic. The KaiC-aa and KaiC-ee mutant proteins that we used in investigations of the binary KaiAC, KaiBC and KaiC-SasA complexes display similar small angle X-ray scattering curves and radii of gyration (**Table 1**). However, the presence or absence of phosphorylation is accompanied by a striking change in the electrostatic surface potential (ESP) on KaiCII that is the site for KaiB binding [26]. That the change is so large is mainly a consequence of the fact that the phosphorylation sites are all situated relatively close to the KaiCII surface. The calculated ESPs for the hypo-phosphorylated form of KaiC (PDB ID 3DVL, computationally modified to have no phosphorylated residues), KaiC-ee (this work), and the hyper-phosphorylated form of KaiC (P-KaiC; PDB 3DVL, computationally modified to have twelve phosphorylated residues) are depicted in **Figure 4C**. The absence and presence of phosphorylation can be expected to affect the conformation of the KaiC structure; however there is also an obvious change from blue (positive) to red (negative) when comparing hypo- and hyper-phosphorylated forms of KaiC. This electrostatic charge difference provides a possible rationalization for KaiB's ability to select between the two forms of KaiC and preferably bind the hyper-phosphorylated form of KaiC.

### SAXS Model of the KaiBC Complex

**Figure 5:**
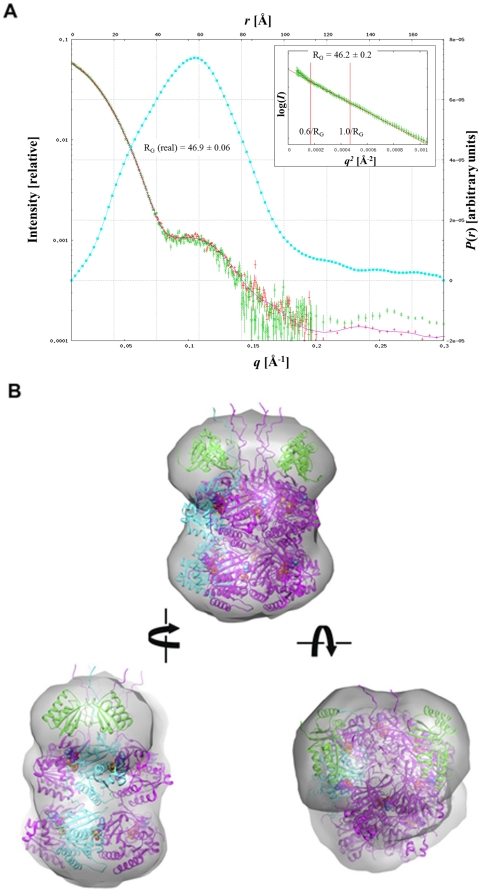
KaiBC complex by SAXS. (A) Experimental scattering curves, pairwise function *P*(r) and Guinier plots (inset) for the KaiBC complex from *S. elongatus* (KaiC-ee mutant). Scattering curves: red with error bars = high concentration, 1.72 mg/mL; green with error bars = low concentration, 1.0 mg/mL; magenta line = FT of *P*(*r*) from GNOM [60]. The cyan curve corresponds to *P*(*r*) from GNOM. Inset: red with error bars = high conc.; green with error bars = low conc.; brown line = Guinier fit of data between the red bars (0.6/*R*_G_ to 1.0/*R*_G_). (B) The SAXS envelope for KaiBC with an EM-based model for KaiBC [26] viewed in three different orientations. The model shows KaiC (magenta and cyan) with two KaiB dimers (green) bound on the CII side. ATP molecules are shown in space filling mode. To model the complete set of KaiC C-terminal peptides emerging from the CII of KaiC, sixfold rotational symmetry was applied to residues 499–519 from subunit A in the wt-KaiC crystal structure [34]. In the panel on the left, fogging was used to obscure the back of the model. The symbols indicate rotations of 90 degrees (left) and 45 degrees (right).

We used native PAGE to determine the optimal conditions for formation of the complex between *S. elongatus* KaiB and KaiC [26]. The KaiC-ee mutant was used in an attempt to stabilize the interactions between the two proteins. Various concentrations of the complex were screened to avoid aggregation in the SAXS experiments. Representative examples of scattering curves, P(r) function and Guinier plots are depicted in **Figure 5A**, and relevant parameters are listed in **Table 1**. Envelopes generated with the program GASBOR [44] reveal a barrel-like shape with a bulge of density along the channel axis on one side of KaiC (**Figure 5B**), as observed for KaiC alone (**Figure 2C**). This allows us to differentiate between the CI and CII halves of the KaiC hexamer since only the CII half has protruding C-terminal peptide tails. The SAXS KaiBC envelope also indicates additional mass on the KaiCII side which we assign to KaiB. Modeling indicates that the additional mass is not very consistent with either a single KaiB tetramer or a pair of tetramers. However, when the earlier EM model of KaiBC with two KaiB dimers [26] is superimposed onto the SAXS envelope, both KaiB dimers are surrounded by SAXS density. Thus, we interpret our SAXS data on KaiBC as consistent with our previously published EM-based model with two KaiB dimers bound to the CII side of KaiC (**Figure 5B**).

In addition to our EM-based model of the KaiBC complex [26], a SAXS-based model of the complex had been reported by others [38]. The quaternary arrangements of the two models differ in that the Akiyama model has a KaiB tetramer modeled into the SAXS envelope such that the central channel of KaiC is covered. However, our combined EM and SAXS interpretation of the KaiBC complex is that two KaiB dimers bind to the KaiCII ring such that the central channel remains open (**Figure 5**).

### Complementarity of EM and SAXS Data for KaiAC and KaiBC

We have produced EM-based models for the binary complexes of KaiAC [34] and KaiBC [26]. The SAXS data presented here for KaiAC and KaiBC is in agreement with the EM-based models and provides additional information regarding the conformationally flexible regions of these complexes. The SAXS envelope for KaiAC supports the concept that the “tethered” state of a KaiA dimer bound to the flexible C-terminal tails of KaiC is the predominant state of the complex in solution. Modeling of the crystal structures of KaiA and KaiC into the SAXS envelope of KaiAC indicates that the distance between KaiA and KaiC barrel is approximately 35 Å, as was previously observed by EM. The SAXS envelope can also be considered consistent with a small percentage of the “engaged” state of KaiA, in which the KaiA dimer interacts directly with the KaiC hexameric barrel.

The KaiBC SAXS envelope shows a subtle bulge of density for the KaiC C-terminal tails on one side of the KaiC hexamer, as is also observed in the SAXS envelope for KaiC alone. No density was observed for the flexible KaiC C-terminal tails in the EM reconstruction. Observation of the KaiC C-terminal tail bulge in the KaiBC SAXS envelope confirms that KaiB binds to the KaiCII side of the KaiC hexamer. We had already come to this conclusion from a combination of moderate resolution EM data and a thorough gel analysis; however the SAXS envelope shows directly that KaiB binds to the KaiCII side of KaiC. The EM structure of KaiBC clearly indicates binding of two KaiB dimers to the KaiC hexamer. Although the KaiBC stoichiometry is not immediately obvious from the KaiBC SAXS envelope alone, the EM-based model with two KaiB dimers is consistent with the SAXS data. Once the flexible C-terminal tails of KaiC are considered, the KaiBC SAXS envelope can no longer be held to be consistent with a tetramer of KaiB binding to KaiC, as previously thought [38].

### Insight into the Ternary KaiABC Complex

KaiA is a two-domain protein with a dimerized C-terminal four-helix bundle domain (C-KaiA [21]–[23]) and an N-terminal domain (N-KaiA) that adopts the fold of a canonical response regulator receiver domain [16] (**Figure S7A**). N-KaiA's primary sequence differs from that of receivers, and is devoid of the conserved Asp that is required for phosphorylation. It is acknowledged that C-KaiA alone is sufficient to enhance the autokinase activity of KaiC *in vitro* [16] and this is by C-KaiA's interaction with the extended C-terminal KaiC peptides [22], [34] (**Figure 3**). Curiously, it was found that KaiA produces a bandshift with a KaiBC complex in which KaiC lacked the C-terminal peptides [19]. Although full-length KaiA and KaiB were not found to interact with each other by native PAGE assays [26], a site-directed spin labeling electron spin resonance analysis revealed a direct, albeit transient interaction between KaiA and KaiB [48]. All of this evidence suggests that KaiA may have a second interaction mode with the KaiBC complex that is distinct from C-KaiA's interaction with the C-terminal KaiC peptides.

**Figure 6:**
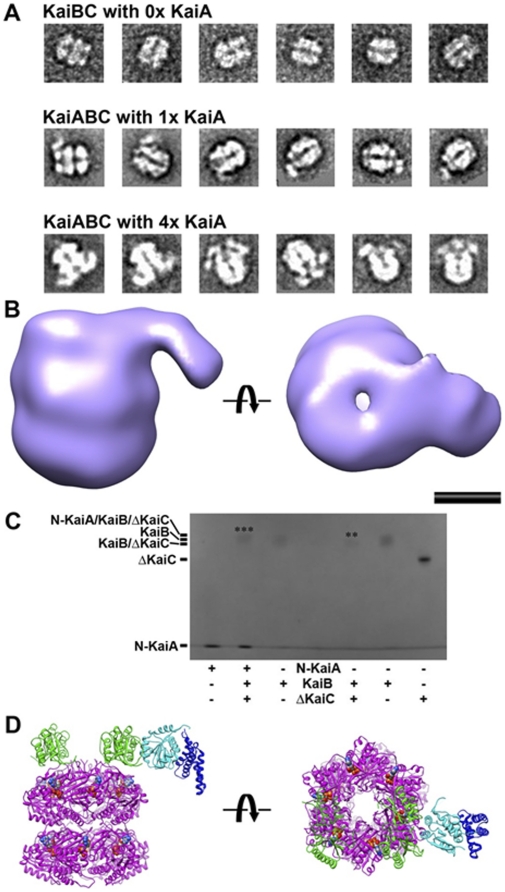
EM model of the ternary KaiABC complex. (A) Negative-stain electron micrographs of the KaiBC complex in the absence of KaiA (0×, top [26]), and in the presence of stoichiometric levels of KaiA (1×, middle) and with 4× the normal concentration of KaiA (bottom). (B) Calculated EM density of the ternary KaiABC complex viewed from the side (left) and along the central channel of the KaiC hexamer (right). The scale bar represents 50 Å. (C) Native PAGE assay of complex formation between KaiB-ΔKaiC and N-KaiA. The KaiB/ΔKaiC band is marked with “**” and is shifted down slightly compared to the KaiB band. The N-KaiA/KaiB/ΔKaiC band is marked with “***” and is shifted up slightly compared to the KaiB band. (D) Model of the KaiABC complex with the flipper-like protrusion, containing the KaiA monomer's N- (cyan) and C-terminal (blue) domains, and viewed from the side (left) and along the central channel in the KaiC hexamer (right). KaiB dimers and KaiC hexamer are colored green and magenta, respectively. The C-terminally truncated form of KaiC (ΔKaiC) is shown. Note the striking resemblance to the EM density in panel B. At the current resolution it is impossible to determine whether N-KaiA engages in an interaction with the KaiB dimer, or with KaiC, or both KaiB and KaiC at their binding interface. The symbols indicate rotations of 90 degrees.

No detailed model of the ternary KaiABC complex has been reported to date. Sorting of negative-stain EM images of Kai complexes formed during the *in vitro* cycling reaction revealed a large complex, presumably the ternary KaiABC complex, which appears similar to the KaiBC complex but with one or two additional lobes of density protruding from the side of the complex (see Figure 1D, class IV in [3]). We hypothesized that the additional side lobes might represent KaiA interacting with the KaiBC complex in its second interaction mode. To test this hypothesis we set up two parallel *in vitro* cycling experiments, one with the normal level of KaiA (1×) and the other with four times the normal level of KaiA (4×). Negative-stain electron micrographs were collected of Kai complexes formed during each cycling reaction. Class average images indicate that in the presence of 4× KaiA there are noticeably more side lobes of density (**Figure 6A**). This supports the idea that the side lobes are KaiA. We also calculated a three-dimensional reconstruction of the class IV complexes from the normal 1× cycling reaction, which displays a prominent KaiA side lobe (**Figure 6B**). The KaiA side lobe density is significantly weaker than that of the KaiC hexamer, indicating a high level of flexibility and making it difficult to recognize any distinctive molecular features of KaiA. Unlike in the binary KaiAC complex where KaiA ‘floats’ above the KaiCII ring of the KaiC hexamer [34] (**Figure 3**), in the ternary complex KaiA appears to be constrained to the side of the KaiBC complex.

When considering how KaiA might interact with KaiBC it is important to note that our EM data [26] and SAXS data (**Figure 5B**) indicate that KaiB interacts with KaiC as a dimer. It is possible that the KaiA binding site on KaiB is obscured in the KaiB tetramer that is normally present in solution (**Figure 2B**), and that the “second mode” KaiA binding site only becomes available when KaiB dimers are bound to KaiC. Alternatively, the KaiB-KaiC interaction may alter the conformation of the KaiCII subunits and expose a new KaiA binding site on KaiC. A third possibility is that the KaiA second mode binding is at a composite site formed at the interface between KaiB and KaiC. To investigate the nature of the KaiA second mode interaction, we conducted native PAGE bandshift assays with N-KaiA and KaiBC complex. These experiments provide support for an interaction between the N-terminal KaiA domain and the binary complex of KaiB and KaiC (**Figure 6C**).

Only a high-resolution structure will ultimately allow insight into the details of the interaction between N-KaiA and KaiBC. However, now that we know that KaiA's interaction with the KaiBC complex is via N-KaiA this opens up the possibility that a monomer of KaiA, and not a dimer, might be involved in the second mode interaction (**Figure 6D**). Furthermore, we postulate that KaiB's antagonism of KaiA action might involve splitting of KaiA dimers, followed by docking of N-KaiA in a ternary complex with KaiBC. This would sequester C-KaiA domains away from the C-terminal tails of KaiC and also prevent KaiA from binding other KaiC hexamers and enhancing their phosphorylation.

### A Three-Dimensional Model of the SasA-KaiC Complex

The His kinase SasA participates in the clock output pathway and teams up with other factors such as RpaA, LabA and CikA to form a relay between the phosphorylation status of the PTO and the global control of transcription [41], [49] (**Figure 1**). Although SasA was initially reported to bind constitutively to KaiC [39], later studies found that KaiC does not just interact rhythmically with KaiA and KaiB but also with SasA [40]. A three-dimensional model of the complex between SasA and KaiC (SasA-KaiC) has remained elusive up to this point. SasA constitutes an EnvZ-like, class I His kinase [39], [50] and exists primarily as a dimer. The binding interaction between SasA and KaiC involves the N-terminal sensory domain (residues 1–97) of SasA (N-SasA) [39]. The sequence identity and similarity between KaiB and N-SasA are approximately 26% and 60%, respectively. It has been noted that their structures have an overall similarity, but they do exhibit distinct features [42] (**Figure S7B,C**). In the same work, it was suggested but not proven that SasA and KaiB might compete for a similar binding site on KaiC.

**Figure 7:**
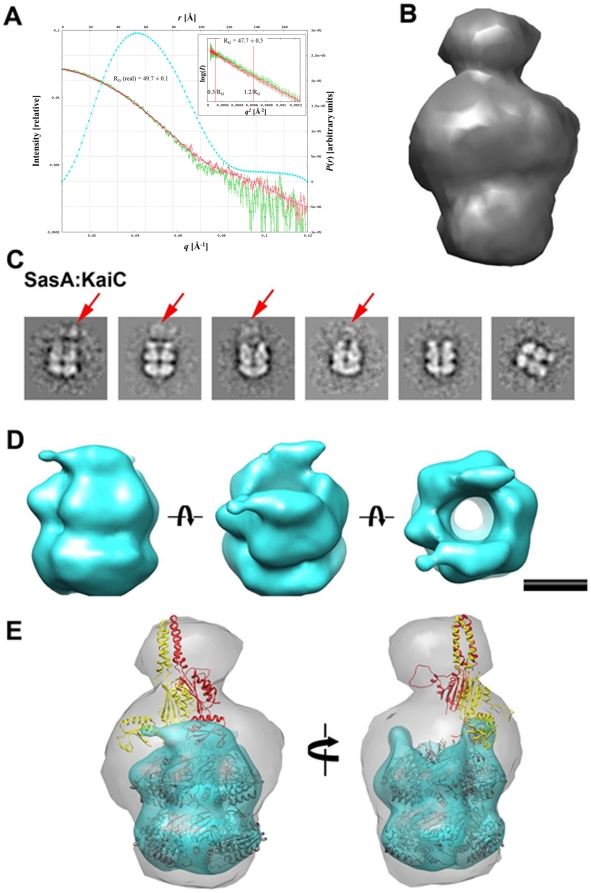
SasA-KaiC complex by SAXS and EM. (A) Scattering curves I(*q*), pairwise function P(*r*) and Guinier plots (inset) for the SasA-KaiC complex (KaiC-ee mutant). Scattering curves: red with error bars = high concentration, 1.71 mg/mL; green with error bars = low concentration, 0.85 mg/mL; magenta = FT of GNOM scan *P*(*r*). The cyan curve corresponds to *P*(*r*) from GNOM [60]. Inset: red with error bars = high conc.; green with error bars = low conc.; brown line = Guinier fit of data between the red bars (0.5/*R*_G_ to 1.2/*R*_G_). (B) SAXS envelope for the SasA-KaiC complex. (C) EM class average images for the SasA-KaiC complex (KaiC-aa mutant); red arrows point to extensive density above a third layer. (D) Calculated EM density for the SasA-KaiC complex viewed from the side, tilted by 45 degrees to show the CII half with the two N-SasA domains bound on either side of the rim, and along the central KaiC channel (left to right). The EM density reveals the location of the N-SasA domain bound to KaiC (the third layer in the images depicted in panel C). The scale bar represents 50 Å. (E) Three-dimensional model of the SasA-KaiC complex with superimposed SAXS envelope (gray) and EM reconstruction (cyan). Only one SasA dimer (red/yellow) is shown for clarity. The symbol indicates a rotation of 45 degrees (panel D) and 90 degrees (panel E).

**Figure 8:**
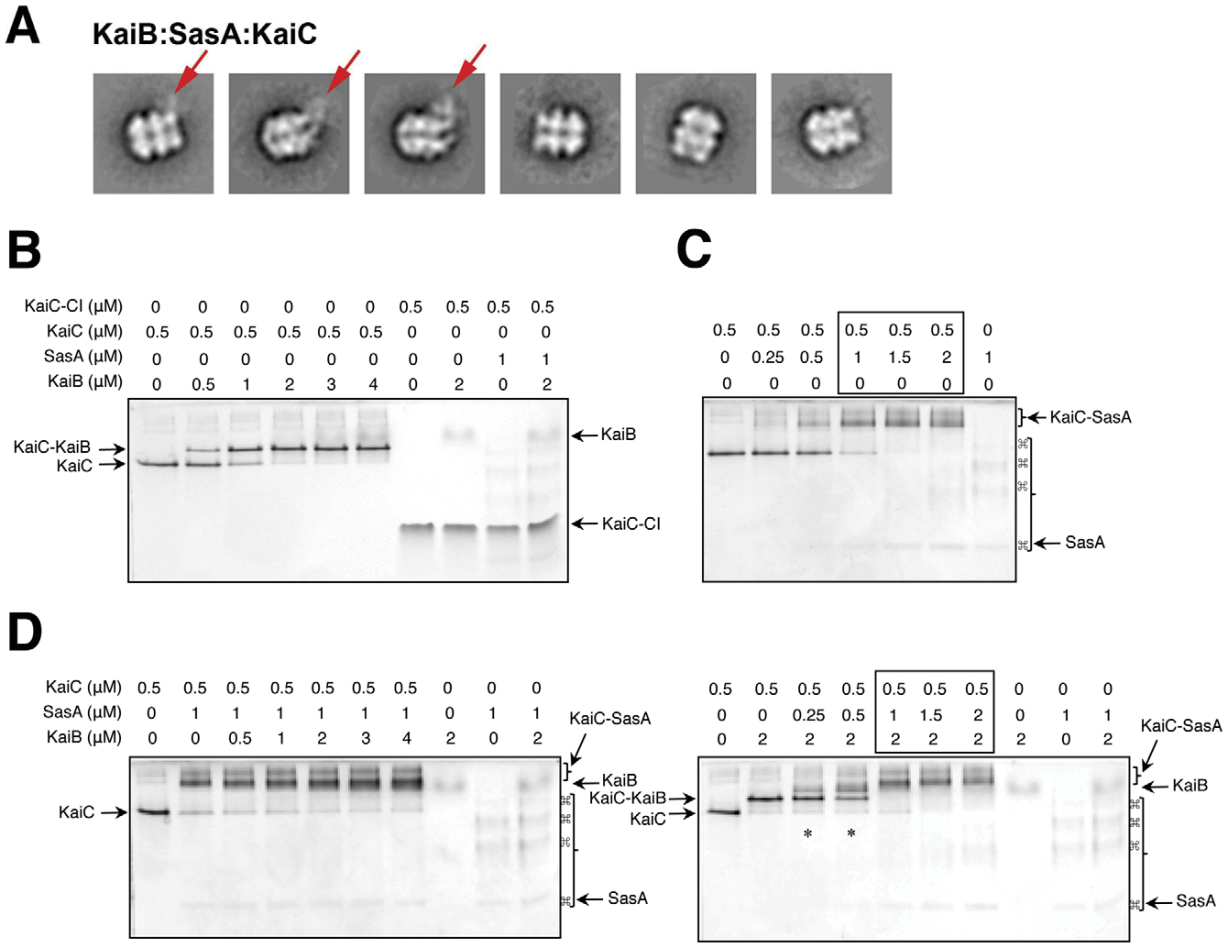
SasA and KaiB compete for the same binding site on KaiC. (A) Negative stain electron micrographs of mixtures of SasA, KaiB and C-terminally truncated KaiC (ΔKaiC); red arrows indicate the location of bound SasA. (B) Native bandshift gel assay for the formation of the binary complex between KaiB and KaiC. Note that neither KaiB nor SasA binds to the KaiCI hexamer (rightmost lanes). Arrows indicate the positions of KaiB, KaiC, KaiBC and KaiCI. (C) Native bandshift gel assay for the formation of the binary complex between SasA and KaiC. Arrows indicate the positions of SasA and SasA-KaiC complex. (D) Competitive binding by SasA and KaiB to KaiC assayed by native PAGE. When increasing amounts of KaiB are added to a pre-existing binary SasA-KaiC complex (gel image on the left), KaiB appears unable to displace SasA. Conversely, when increasing amounts of SasA are added to a pre-existing binary KaiB-KaiC complex (gel image on the right), SasA infiltrates the complex and a band for the ternary complex appears (marked by an asterisk). Upon further increasing the SasA concentration, KaiB is completely displaced, resulting in the formation of the binary SasA-KaiC complex (compare boxed lanes in panel D to those boxed in panel C). Not only do KaiB and SasA interact with the same KaiCII regions, but binding between SasA and KaiC is considerably more tight than binding between KaiB and KaiC.

Binding between SasA and either KaiC-ee or KaiC-aa was established by native PAGE (**Figure S8**). Complex formation was observed with both KaiC mutants, which were designed as mimics of the hyper- and hypo-phosphorylated states, respectively. SAXS experiments were conducted with the SasA:KaiC-ee complex and representative experimental data are shown in **Figure 7A**. The envelope generated with the program GASBOR [44] for SasA-KaiC reveals a mushroom-like protrusion along the sixfold axis of the KaiC hexamer (**Figure 7B**). There is no bulge for the KaiC C-terminal tails on the opposite end of the hexamer, suggesting that SasA binds to the KaiCII side of KaiC, as does KaiB. The KaiC C-terminal tails presumably are present, along with SasA, within the mushroom-like protrusion. The suggestion of SasA binding to the KaiC CII domain is supported by an EM competition experiment between SasA and KaiB for binding to KaiC (**Figure 8**), together with our previous experimental data showing binding of KaiB to the CII domain [26].

Negative stain EM studies were performed with the SasA:KaiC-aa complex. A set of ∼3,000 particle images were classified by the EMAN algorithm refine2d.py to generate 50 classes [51]. Approximately half of the class average images show additional density assigned to SasA at one end of the KaiC hexameric barrel (**Figure 7C**). The additional SasA density includes a well resolved third layer on top the KaiC barrel, similar to that observed for KaiB in the KaiBC complex [26], as well as weaker density in variable positions over the triple-layer structure. We interpret the third well-resolved layer of density as corresponding to the SasA N-terminal sensory domain, which is known to bind KaiC, and the additional blurred density as corresponding to the SasA dimerization/H-box and catalytic domains. A three-dimensional reconstruction of SasA:KaiC-aa at ∼23 Å resolution was generated with the EMAN refine command (**Figure 7D**). The EM structure of SasA-KaiC shows a recognizable hexameric barrel shape for KaiC and a third layer of density with two apparent binding sites for the SasA N-terminal domain on either side of the KaiC central channel. The shape of the SasA-KaiC EM structure is reminiscent of that observed for KaiBC with two KaiB dimers bound to a KaiC hexamer on either side of the central channel [26]. Essentially no density is observed in the SasA-KaiC EM structure for the SasA dimerization and catalytic domains, which presumably correspond to the blurred density in the class average images.

NMR solution structures for the sensory, dimerization/H-box and catalytic domains of SasA or close homology models (**Figure S9A–C**) were combined to generate a three-dimensional model of the full-length SasA dimer. The published SAXS model of the class I PrrB His kinase [52] served as a guide for the overall domain organization (**Figure S9D**). The linkers between individual SasA domains are quite long and can be expected to lead to a variable overall domain organization. Therefore the domains within the SasA dimer model were adjusted as rigid bodies during the fitting within the SAXS and EM envelopes (**Figure 7E**).

Combining the model of the full-length SasA dimer with the SasA-KaiC SAXS envelope and the SasA-KaiC EM structure enabled us to build a reasonable three-dimensional model for the complex (**Figure 7E**). The EM density is consistent with monomers of the SasA N-terminal domain binding to the KaiC hexameric barrel on opposite sides of the central channel. Modeling with the full-length SasA dimer makes it seem unlikely that the two opposing SasA N-terminal domain binding sites would be occupied by two domains from the same SasA dimer. Rather the EM density is more consistent with having the second N-terminal domain in each of two bound SasA dimers hover near KaiC but not actually bind to KaiC. These unbound SasA N-terminal domains could potentially bind to other KaiC hexamers and lead to chaining of SasA-KaiC complexes. Some aggregation for the SasA-KaiC sample was observed by SAXS.

The individual SasA domains as docked within the SAXS envelope of the SasA-KaiC complex were optimized with several rounds of rigid-body refinement, eventually yielding a good fit such that the SAXS envelope covers two full-length SasA dimers, with two of the four N-terminal sensory domains occupying binding sites on opposite sides of the KaiCII rim. The volume of the SasA-KaiC SAXS envelope is consistent with binding of two SasA dimers per KaiC hexamer, although only one SasA dimer is shown for clarity in **Figure 7E**. In our SasA-KaiC model the distal protrusion in the SAXS envelope corresponds to the α-helical bundles in the central domain that stabilize the SasA dimer. The somewhat loose fit of the model in the envelope for the distal protrusion, as well as the corresponding blurred density in the EM class average images, suggests that the linker between the SasA N-terminal domain that binds KaiC and the SasA central α-helical domain must be quite flexible.

Superimposition of the SAXS envelope and EM structure provided guidance for building a three-dimensional model the SasA-KaiC complex. This model is intended to show the overall stoichiometry and placement for domains of SasA and KaiC. Neither the SAXS envelope nor the moderate resolution EM structure can provide insight into the orientation of the N-terminal SasA sensory domain relative to the KaiCII ring. This will require a higher resolution structural analysis, perhaps with designed fragments of SasA and KaiC. The individual SasA domains as docked within the SAXS envelope of the SasA-KaiC complex were optimized with several rounds of rigid-body refinement, eventually yielding a good fit such that the SAXS envelope covers two full-length SasA dimers, with two of the four N-terminal sensory domains occupying binding sites on opposite sides of the KaiCII rim.

### A Competition Experiment Shows that SasA and KaiB Bind to the Same Side of KaiC

In order to confirm that SasA and KaiB compete for binding sites on the same side of the KaiC hexamer an EM-based competition experiment was performed. Mixtures were formed with a C-terminally truncated form of KaiC, KaiC-Δ489 [34], which is known to form stable complexes with KaiB, together with wt KaiB and wt SasA. Negative stain electron micrographs were collected of the mixture. A set of 6,013 particle images were classified by the EMAN refine2d.py algorithm to generate 25 classes [51] (**Figure 8A**). The class average images resemble those of SasA:KaiC-aa (**Figure 7C**) and include some images of triple layer complexes resembling KaiBC [26] and some triple layer complexes with additional weak density hovering over the triple layer structure. None of the class average images of the KaiC-Δ489-KaiB-SasA mixture show a quadruple layer structure. We also used native PAGE to independently assay KaiB-KaiC (**Figure 8B**) and SasA-KaiC binding (**Figure 8C**) and a potential competition between SasA and KaiB for binding to KaiC (**Figure 8D**). We carried out two different experiments. In the first, increasing amounts of KaiB were added to mixtures of SasA and KaiC, and in the second, increasing amounts of SasA were added to mixtures of KaiB and KaiC, and the resulting complexes analyzed. While KaiB is unable to displace SasA from KaiC (**Figure 8D**, left panel), SasA infiltrates the KaiB-KaiC complex, initially resulting in formation of a ternary complex (**Figure 8D**, right panel). Upon addition of further SasA, the protein completely displaces KaiB under formation of the binary SasA-KaiC complex. Thus, the native PAGE experiments demonstrate that: (i) Neither KaiB nor SasA binds to KaiCI (**Figure 8B**), (ii) the two proteins competitively bind to the same KaiCII region, and (iii) binding between SasA and KaiC is considerably more tight than binding between KaiB and KaiC. Overall, SAXS, EM, and gel analyses all indicate that SasA and KaiB compete for overlapping binding sites on the KaiCII side of KaiC. Further support for this conclusion is provided by the earlier observation of abnormally dominant complex forms of KaiC in *kaiB*-inactivated *S. elongatus* strains that co-fractionate with SasA [40].

## Discussion

The discovery that the *S. elongatus* oscillator can be assembled *in vitro* from three proteins in the presence of ATP [2] had to fill anybody studying molecular clocks with a renewed sense of awe of nature's ingenuity. To biochemists and biophysicists this three-component PTO offers a multitude of challenges, ranging from cooperativity among KaiC subunits and ATPase energy transfer to reaction mechanisms and the molecular origins of temperature compensation. Many efforts have been directed at the core protein KaiC and its kinase, phosphatase and ATPase activities (reviewed in [4], [5], [53]). However, just as mechanical clocks need an escapement in addition to the main spring and gear train, the cyanobacterial clock doesn't tick with KaiC alone. Therefore, understanding how KaiA and KaiB proteins interact with KaiC to drive the oscillator and maintain a period of close to 24 hours is of fundamental importance.

We previously employed negative-stain and cryoEM to gain insight into the protein-protein interactions in the binary KaiAC [34] and KaiBC complexes [26]. Here, we have used a combination of SAXS, EM, X-ray crystallography and PAGE to further characterize these two complexes, shed light on the interaction between KaiC and the His kinase SasA that participates in the clock output pathway, and produce the first three-dimensional model of the ternary KaiABC complex. In spite of the different sample environments, the new SAXS and earlier negative stain EM envelopes of the KaiAC complex are quite similar and indicate the same stoichiometry, namely binding of a single KaiA dimer to a KaiC hexamer. The SAXS envelope of KaiAC reveals a predominant orientation of the KaiA dimer relative to the KaiCII surface that is consistent with the so-called ‘tethered’ form of the complex based on EM [34]. In the ‘tethered’ form KaiA resides at some distance (∼35 Å) from CII, and is tethered to the hexameric barrel via a C-terminal KaiC peptide [22] (**Figure 3**). Besides the tethered arrangement, the EM density was also indicative of a configuration in which KaiA and KaiC are more tightly spaced (the so-called ‘engaged’ form of the complex [34]). In this state, the C-terminal domain of a KaiA monomer may contact a secondary binding site on KaiC that includes the ATP binding cleft between subunits, thus potentially prolonging the ATP residence time [34].

We show that the quaternary structures of the individual Kai proteins in solution as determined by SAXS match those in the solid (crystalline) state - KaiA dimer, KaiB tetramer and KaiC hexamer (**Figure 2**). In the SAXS analysis of KaiC alone, C-terminal peptides (residues 489–519) from the six subunits give rise to a dome-shaped density on the KaiCII side (**Figure 2C**). A similar feature is also present in the KaiBC complex. This SAXS result confirms the idea that KaiB binds to the CII side of KaiC. In our analysis after docking KaiC the remaining density in the SAXS envelope of the KaiBC complex is best modeled as two KaiB dimers (**Figure 5B**), rather than one or two KaiB tetramers. A KaiB tetramer lying down on the KaiCII dome would act as a lid for the central channel, and this is inconsistent with EM images of the complex that reveal a central opening [26]. Also, neither the earlier EM data [26] nor the SAXS envelope of the KaiBC complex provide support for a binding mode with a portion of one or two KaiB tetramers extending beyond the edge of the KaiCII ring. Our EM-based model for the KaiBC complex [26], with two KaiB dimers bound to the KaiC hexamer, agrees well with the KaiBC SAXS data.

The change in quaternary structure for KaiB upon binding to KaiC is unanticipated, and earlier considerations of the role played by the particular charge distribution in the KaiB tetramer in terms of the interaction with KaiC and as a promoter of dephosphorylation [24], [25] require revision. This is because the inter-dimer interfaces of the KaiB tetramer now have to be taken into account as well when considering the possible makeup of the KaiB-KaiCII binding interface. The SAXS and EM envelopes at the current resolutions are not sufficient to settle the orientation of the KaiB dimer, but there are two additional sources of information that should be helpful in this regard. First, it has been established that the KaiB C-terminal tail, rich in acidic residues, is important for function [25]. Therefore, we would expect the tail to be directed toward KaiC where it may help drive subunits apart and interfere with binding of ATP, the only other molecular entity in the region that features a similarly negative charge as the KaiB tail. Secondly, the ESP of the dome-shaped CII end of KaiC is positively polarized in the hypo-phosphorylated (T432/S431 = TS) state. The transition to the hyper-phosphorylated state goes along with the appearance of more negatively polarized patches (**Figure 4C**). This likely constitutes the signal for KaiB binding and its ESP can be expected to be complementary to that of KaiCII in the pTpS phosphorylation state. In the subsequent TpS state in which T432 has been dephosphorylated, KaiC exhibits a slightly expanded conformation [47] compared to the compact pTpS state seen in the crystal structure [10]. If KaiB inserts its C-terminal tail between two subunits the ATP binding cleft may open up, thus further loosening the association between them and prying the monomers apart and initiating monomer exchange. It is reassuring in terms of the three-dimensional structure of the KaiBC complex that both EM and SAXS are consistent with a model involving binding of two KaiB molecules to KaiCII (**Figure 5B**).

Our combined SAXS- and EM-based model of the SasA-KaiC binary complex reveals binding of two N-terminal sensory domains of the His kinase along the rim region of KaiCII, in a similar spot to that occupied by KaiB (**Figures 7D,E** and **8**). The two molecules (SasA and KaiB) appear to compete for a similar binding site on KaiC, consistent with their shared ability to sense the phosphorylation status of the latter. It has been noted that KaiB and SasA display common features as far as their ESPs are concerned [42]. We can envision KaiB binding on one side of the KaiCII rim and SasA occupying the other side, while KaiA remains attached to a C-terminal KaiC peptide, in line with the binding of SasA to KaiC over the entire 24-hour period of the *S. elongatus* PTO [39]. A clear difference between SasA and KaiB binding is that SasA probes the KaiC phosphorylation status using a monomeric sensor. There is presently no evidence, despite similar folds of KaiB and N-SasA (**Figures S7B** and **S7C**, respectively) that would suggest that KaiB binds as a monomer. A further difference between the two proteins relates to the C-terminal region; SasA lacks the C-terminal tail rich in acidic residues that is a hallmark of KaiBs from a host of cyanobacterial strains including *S. elongatus*. Instead the C-terminal extension of the SasA sensory domain serves as a linker to the dimerization motif.

That the PTO lies at the heart of the *S. elongatus* circadian clock is underscored by the aberrant behavior of the KaiC-ee double mutant that was used to mimic the hyper-phosphorylated state ([6] and cited refs.). The crystal structure of KaiC-ee reveals a new phosphorylation site at S320 and an altered environment of E432 compared to pT432 in the crystal structure of wt-KaiC (**Figure 4A**). In the wild type structure a salt bridge between phosphothreonine and R385 across the subunit interface is likely of importance for the tight association of KaiCII domains from the six subunits in the pTpS state. By comparison in the KaiC-ee structure, E432 undergoes a shift and the carboxylate moiety is cradled by two serines (S379 and S381) and a threonine (T415) from the adjacent subunit. R385 is nearby but the distance between the Nε atoms of R385 and the carboxylate oxygens of E432 exceeds 5 Å in all cases. In the KaiCI half, the residues corresponding to E432 (wt T432) and E431 (wt S431) are E198 and E197 and T79 takes the place of S320 in KaiCII (**Figure S10**). KaiCI shows ATPase activity but lacks the kinase and phosphatase activities harbored by the CII half. Although the distance between T79 and the γ-phosphate of ATP in KaiCI is similar to the corresponding distance for S320 in KaiCII (12.35 vs. 12.41 Å, respectively; A subunit, S/T Cα), T79 is clearly not phosphorylated in any of the six subunits. This absence of the phosphotransfer in the CI half reflects subtle differences between the CI and CII subunit interfaces, as well as the CI and CII domain sequences, conformations and dynamics. What we learn from the KaiC-ee structure is that mutations that are intended to mimic a particular native state (hyper-phosphorylated; pTpS) can affect structure and activity in unexpected ways. In this case, the different charges and chemistries of the glutamate and phosphothreonine side chains lead to a new phosphorylation site (**Figure 4A,B**).

Our gel analysis with N-KaiA, KaiB, and C-terminal truncated KaiC, KaiC-Δ489, indicates a new interaction between the N-terminal KaiA domain and either one or both of the other Kai proteins in the ternary KaiABC complex (**Figure 6**). The EM structure of the KaiABC complex is consistent with binding of KaiA to the KaiBC complex and with KaiA jutting out from the side of KaiBC. Although the EM study was performed with wild type KaiC, the gel analysis indicates that the C-terminal tails of KaiC are not necessary for this new interaction with KaiA. The binding geometry we propose for the KaiABC complex (**Figure 6D**) suggests that perhaps KaiA is no longer holding on to a KaiC C-terminal peptide. Rather N-KaiA is bound to KaiBC via a new, as yet uncharacterized, interaction site. In terms of function, KaiB may influence the action of KaiA by preventing KaiA from adopting the ‘engaged’ configuration as we proposed before [26]. In addition, KaiB may effectively sequester KaiA in this new binding configuration and therefore disable it from contacting other KaiC molecules and boosting their phosphorylation [18], [19].

**Figure 9:**
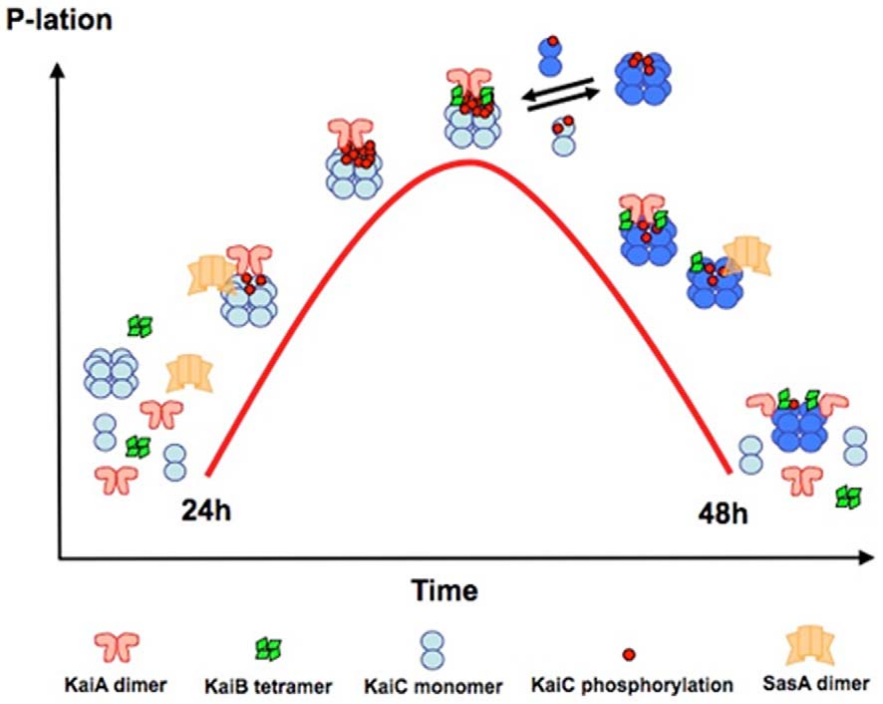
Schematic illustration of PTO composition, protein-protein interactions and changes in the quaternary structures of Kai proteins in the cyanobacterial oscillator over a single 24-h period. Binding of a KaiA dimer via its C-terminal domains causes a change in the KaiCII hexamer from the hypo- to the hyper-phosphorylated state, eventually triggering binding of a KaiB tetramer in the form of two separate dimers on either side of the KaiCII ring. KaiB binding is accompanied by KaiC subunit exchange and SasA dimer interacts with the PTO in a circadian fashion and competes with KaiB for binding to KaiC. Formation of the KaiBC complex results in release of the KaiA dimer from the C-terminal KaiCII tail and subsequently in reattachment via its N-terminal domain, leading to KaiA sequestration in a stable ternary KaiABC complex at the final stage of the clock cycle.

Our analysis of the protein-protein interactions in the *S. elongatus* core circadian oscillator using a hybrid structural approach reveals a more dynamic interplay of the KaiA, KaiB and KaiC proteins than was hitherto anticipated (**Figure 9**). Earlier research had uncovered subunit exchange between KaiC hexamers during the dephosphorylation phase of the clock cycle and linked this behavior to maintenance of a robust amplitude by the cyanobacterial timer [3], [7]. We now demonstrate that KaiA and KaiB also undergo changes in their quaternary structure over the daily period. KaiB is known to form a tetramer in the solid state and in solution, but our new SAXS data confirm that it binds to KaiC in a dimeric form. KaiA binds the C-terminal tail of a KaiC subunit above the dimer interface of its C-terminal domains to increase KaiC phosphorylation during the first half of the clock cycle. KaiB associates with KaiC once the hyper-phosphorylated stage is reached, thus triggering KaiC subunit exchange and dissociation of the bound KaiA dimer. KaiA then reattaches itself to the KaiBC complex, but unlike during the initial phase of the clock cycle, binding now involves the N-terminal KaiA domain (**Figure 6C,D**).

To the best of our knowledge, this work represents the first example of an extensive SAXS and EM comparative structural analysis of a protein complex, SasA-KaiC. This study supports the concept that hybrid methods, including X-ray crystallography, high-field NMR, SAXS and EM can provide complementary information for challenging and conformationally flexible macromolecular assemblies. Future work will be directed at higher resolution dissection of the protein-protein interactions in the KaiABC clock and its output pathway using cryoEM and X-ray crystallography. Without such high-resolution information a proper understanding of the mechanisms by which KaiA and KaiB modulate KaiC's kinase and phosphatase activities and readout will remain obscure.

## Materials and Methods

### Enzyme expression and purification

The *S. elongatus* GST-KaiA, GST-KaiB and GST-KaiC fusion proteins were expressed in *E. coli* [BL21, DE cell line (Invitrogen)] and purified as described in [3], [29]. The *S. elongatus* KaiC protein with a C-terminal (His)_6_-tag was produced in *E. coli* as previously described [8], [10] and purified by affinity and gel filtration chromatography. Site-directed mutagenesis (S431A/T432A = KaiC-aa, T426A/S431A/T432A = KaiC-aaa, T426A/S431E/T432E = KaiC-aee, S431E/T432E = KaiC-ee, S431D-KaiC, and E318A-KaiC) was performed with the QuikChange® XL site-directed mutagenesis system (Stratagene, La Jolla, CA) and all mutant proteins were expressed as GST-fusions following the protocol used with wt-KaiC. Mutant proteins were purified by metal affinity chromatography (TALON IMAC resin, BD Biosciences Clontech) and then by gel filtration chromatography (Sephacryl S-300 HR resin, Amersham Biosciences). All proteins were analyzed by tryptic digestion followed by MALDI-TOF mass spectrometry. The solution of the purified KaiC-ee mutant protein was concentrated to ca. 10 mg/mL and ATP in the buffer was replaced with ATPγS by ultrafiltration for crystallization.

### X-ray crystallography

Crystals of the KaiC-ee mutant protein were grown under conditions similar to those established earlier for wt-KaiC from *S. elongatus* [10]. Following mounting in a nylon loop and cryo-protection in 25% glycerol, diffraction data were collected on the 21-ID-F beamline of the Life Sciences Collaborative Access Team at the Advanced Photon Source (APS; Argonne National Laboratory, Argonne, IL) using a wavelength of 1.000 Å. Data were integrated and scaled with HKL2000 [54] and the initial orientation of KaiC was established by molecular replacement with the program CNS [55], using as the search model the wt-structure (PDB ID 3DVL [30] minus water and Mg^2+^ and with residues pS431 and pT432 mutated to alanine). Rigid body refinement was followed by cycles of positional refinement with CNS. Manual rebuilding was performed with the programs TURBO [56] and COOT [57]. Side chains of residues 431 and 432 were added and their conformations gradually adjusted in the six subunits, followed by further rounds of positional and isotropic B-factor refinement and addition of solvent molecules. Selected crystal data, data collection and refinement parameters are summarized in **Table S1**. Illustrations were generated with the program CHIMERA [45].

### Gel electrophoresis

Native PAGE was carried out on a PhastSystem (Pharmacia LKB) using PhastGel Gradient 4–15% or 8–25% gels and PhastGel Native Buffer Strips (Amersham Biosciences). The gels were stained with 0.1% PhastGel Blue R solution in 10% acetic acid and 30% methanol and destained with 30% methanol and 10% Acetic acid. SDS-PAGE was performed using the mini-gel system from Bio-Rad and ready-made 10% Tris gels (Bio-Rad). Bio-Safe Coomassie G250 solution was used for staining.

### *In vivo* rhythm experiments

A luciferase reporter strain was made by introducing a gene fusion kaiBCp::luxAB into the neutral site (NS) I of *S. elongatus* PCC 7942, in which the expression of the *Vibrio harveyi* luciferase structure genes luxAB is driven by the promoter of the KaiBC genes. Mutation of KaiC at 426, 431, and 432 was performed by site-directed mutagenesis, and the clock-controlled rhythms in wild type or mutant strains were monitored by real-time measurement of luminescence as described previously [33].

### ^32^P-labeled KaiC phosphorylation *in vitro*

*In vitro* phosphorylation of KaiC proteins by using [γ-^32^P]ATP was performed as described previously [33]. Briefly, purified proteins (200 ng/µL) of wild-type KaiC (TST) or mutant KaiCs (aaa or aee) were incubated either at 4 or 30°C in 20 mM Tris-HCl, 150 mM NaCl, 5 mM MgCl2, 0.5 mM EDTA, 1 mM non-radioactive ATP and 0.4 µCi/µL [γ-^32^P]ATP. At each time point, 10 µl aliquots were removed, mixed with10 µL of 2× SDS-PAGE loading buffer, and stored at −20°C. After heating at 100°C for 10 min, the samples were processed for SDS-PAGE and autoradiography.

### SAXS data collection, processing and model building

All small angle X-ray scattering data were collected on the SAXS/WAXS setup located at the 5-ID-D beamline of the DND-CAT synchrotron research center, Advanced Photon Source, Argonne National Laboratory (Argonne, IL). 1.2398 Å radiation was selected from the APS Undulator A spectrum using a Si-111 monochromator, with harmonic rejection provided by a 1∶1 horizontally focusing mirror, and collimated to 0.3×0.3 mm with a series of polished slits. The SAXS detector was a Mar-USA 162 mm CCD and covered the momentum transfer range 0.005<*q*<0.20 Å^−1^, where *q* = 4π sinθ/λ (2θ is the scattering angle). The WAXS detector was a custom Roper CCD and covered 0.19<*q*<1.8 Å^−1^. Optimal conditions for the various binary complexes were established using native PAGE and all solutions were assessed with light scattering. Scattering curves for KaiA, KaiB, KaiC and the three binary complexes at three concentrations as well as all individual buffer solutions were recorded at 10 or 20°C. After averaging and normalization by the sample concentration (protein concentrations were between 0.5 and 2.0 mg/mL), scattering due to the buffer alone was subtracted and the data condensed and noise removed [58]. Data in the low- and high-angle ranges were merged and cropped to various ranges of *q*. The innermost portions of the scattering curves were used for fitting to the equation I(*q*) = I(0) exp(−4π^2^R_G_^2^ *q* ^2^/3), where I(0) is the forward scattering intensity at *q* = 0 and R_G_ is the radius of gyration [59]. Values for R_G_ were extracted from the Guinier plot log{I(*q*)} vs. *q* ^2^ or the pair distribution function P(r) using the GNOM package [60].

### *Ab initio* envelope calculations

Molecular envelopes were calculated with the programs DAMMIN and GASBOR along with the averaging package DAMAVER [44], [61], using the SIBYLS SAXS ATSAS program (http://bl1231.als.lbl.gov/saxs_protocols/saxs_programs.php [62]). Twenty averaged *ab initio* models were calculated and scored before selecting the most typical one. No symmetry restraints were applied in any of the shape reconstructions. The resulting NSD values (**Table 1**) revealed a good similarity in shape for models of Kai proteins alone and the binary KaiC complexes, representative examples of which are depicted in the various figures. Three-dimensional structures of full-length proteins based on crystal structures (KaiA, KaiB and KaiC) or domains based on solution NMR (SasA) were built into the SAXS envelopes manually and optimized via rigid body refinement in some cases using CHIMERA [45].

### Electron microscopy and image processing

Complexes between KaiC-aa and SasA were confirmed by native PAGE prior to performing negative stain EM analysis. Protein was diluted to give good individual particle dispersion of the complex on glow discharged solid carbon supports on 400 mesh Cu foils. Uranyl formate at a concentration of 0.75% was used as the contrast agent. Micrographs were collected on a FEI Tecnai T12 microscope operating at 120 kV with a magnification of 68,000× and a defocus range between −1 and −1.5 µm on a Gatan US1000 2k×2k CCD camera. Digital micrographs were binned by a factor of 2 prior to image processing. A total of 2,955 particles were selected with the EMAN Boxer routine [51]. Principal component analysis and classification with EMAN Refine2d.py resulted in 50 class average images. Orientational parameters were determined and a three-dimensional image reconstruction was calculated using C1 symmetry with the EMAN Refine routine. The final reconstruction was generated from 2,786 particle images and has an estimated resolution of 23.5 Å at the Fourier Shell Correlation (FSC) 0.5 threshold.

The EM reconstruction of KaiABC is based on the meta class IV images selected from a negative stain EM dataset of complexes imaged during the *in vitro* oscillation cycle described in [3]. The full data set contained 69,749 particle images classified with EMAN Regine2d.py into 1750 class sum images and then meta-sorted into meta classes I–IV. The resolution of the KaiABC reconstruction is ∼30 Å.

### Crystallographic coordinates

Final coordinates and structure factors for the crystallographic model of the *S. elongatus* KaiC-ee mutant protein have been deposited in the Protein Data Bank (http://www.rcsb.org): PDB ID code 3S1A.

## Supporting Information

Figure S1 Scattering curves I(q), pairwise function P(r) and Guinier plots (inset) for S. elongatus KaiA. Scattering curves: red with error bars (from GNOM) = high concentration, 2.1 mg/mL; green with error bars (from GNOM) = low concentration, 0.85 mg/mL; magenta line = FT of GNOM scan P(r). The cyan curve corresponds to P(r) from GNOM (Svergiun, 1992). Inset: red with error bars = high conc., 2.1 mg/mL; blue with error bars = medium conc., 1.5 mg/mL; green with error bars = low conc., 0.85 mg/mL; brown line = Guinier fit of data between the red bars (0.4/RG to 1.2/RG).
(TIF)

Figure S2 Scattering curve I(q), pairwise function P(r) and Guinier plot (inset) for S. elongatus KaiB. Scattering curve: red with error bars, concentration 1 mg/mL; magenta line = FT of GNOM scan P(r). The cyan curve corresponds to P(r) from GNOM (Svergun, 1992). Inset: red with error bars = conc. 1 mg/mL; brown line = Guinier fit of data between the red bars (0.7/RG to 1.2/RG).
(TIF)

Figure S3 Scattering curves I(q), pairwise function P(r) and Guinier plots (inset) for S. elongatus KaiC. Scattering curves: red with error bars (from GNOM) = high concentration, 0.95 mg/mL; blue with error bars (from GNOM) = medium concentration, 0.80 mg/mL; green with error bars (from GNOM) = low concentration, 0.66 mg/mL; magenta line = FT of GNOM scan P(r). The cyan curve corresponds to P(r) based on the medium concentration from GNOM (Svergun, 1992). Inset: red with error bars = high conc., 0.95 mg/mL; blue with error bars = medium conc., 0.80 mg/mL; green with error bars = low conc., 0.66 mg/mL; brown line = Guinier fit of data between the red bars (0.5/RG to 1.2/RG).
(TIF)

Figure S4 Scattering curves I(q), pairwise function P(r) and Guinier plots (inset) for the S. elongatus KaiAC complex (KaiC-aa mutant). Scattering curves: red with error bars = high concentration, 2.1 mg/mL; blue with error bars = medium concentration, 1.6 mg/mL; green with error bars = low concentration, 1.1 mg/mL; magenta line = FT of GNOM med conc. P(r). The cyan curve corresponds to P(r) from GNOM (Svergun, 1992). Inset: red with error bars = high conc. (2.1 mg/mL); blue with error bars = medium conc. (1.6 mg/mL); green with error bars = low conc., 1.1 mg/mL; brown line = Guinier fit of data between the red bars (0.4/RG to 0.9/RG).
(TIF)

Figure S5 Quality of the final crystallographic model for the KaiC-ee mutant. Fourier sum (2Fo-Fc) density contoured at the 1σ level around (A) residue pS320 in the F subunit (phospho-serine; upper right), and (B) around E431 (lower right) and E432 (upper right) in the A subunit.
(TIF)

Figure S6 Effect of Glu substitution of KaiC P-sites on the PkaiBC-driven rhythm. Following a dark synchronization, luminescence was measured in reporter strains expressing either wt-KaiC (KaiCWT) or the KaiC-ee and T426E mutants (KaiCS431E/T432E and KaiCT426E, respectively).
(TIF)

Figure S7 Comparisons between the three-dimensional folds (N-terminus blue to C-terminus red) of (A) N-KaiA [S. elongatus; PDB 1R8J; X-ray (Ye et al., 2004)], (B) KaiB [T. elongatus; PDB ID 2QKE; X-ray (Pattanayek et al., 2008)], and (C) N-SasA (S. elongatus; PDB ID 14TY; NMR (Vakonakis et al., 2004)].
(TIF)

Figure S8 Native PAGE assays for complex formation between either KaiC-ee (left) or KaiC-aa (right) and full-length SasA.
(TIF)

Figure S9 Three-dimensional structures and models of His kinase domains. (A) N-terminal sensory domain of S. elongatus SasA [PDB ID 1T4Y; NMR (Vakonakis et al. 2004)], (B) dimerization domain harboring the His phosphorylation site of E. coli EnvZ [PDB ID 1JOY; NMR (Tomomori et al., 1999)], and (C) the catalytic domain of E. coli EnvZ with bound ADP[PDB ID 1BXD; NMR (Tanaka et al., 1998)]. (D) Model of the full-length SasA dimer, viewed perpendicularly to the molecular dyad, and rotated by 90° and viewed along the molecular dyad. Linker regions are highlighted in black.
(TIF)

Figure S10 Sequence alignment and secondary structures of S. elongatus KaiCI (upper line) and KaiCII (lower line).
(TIF)

Table S1 Selected crystal data, X-ray data collection and refinement parameters for the crystal structure of *S. elongatus* KaiC-ee*^a^*.
(DOCX)

References S1 Supplementary References.
(DOCX)
