# Supplementary figures and images for: Combined SAXS/EM Based Models of the S. elongatus Post-Translational Circadian Oscillator and its Interactions with the Output His-Kinase SasA
Source: PLoS One. 2011 Aug 24;6(8):e23697. doi: 10.1371/journal.pone.0023697 (PMC3161067; doi:10.1371/journal.pone.0023697)

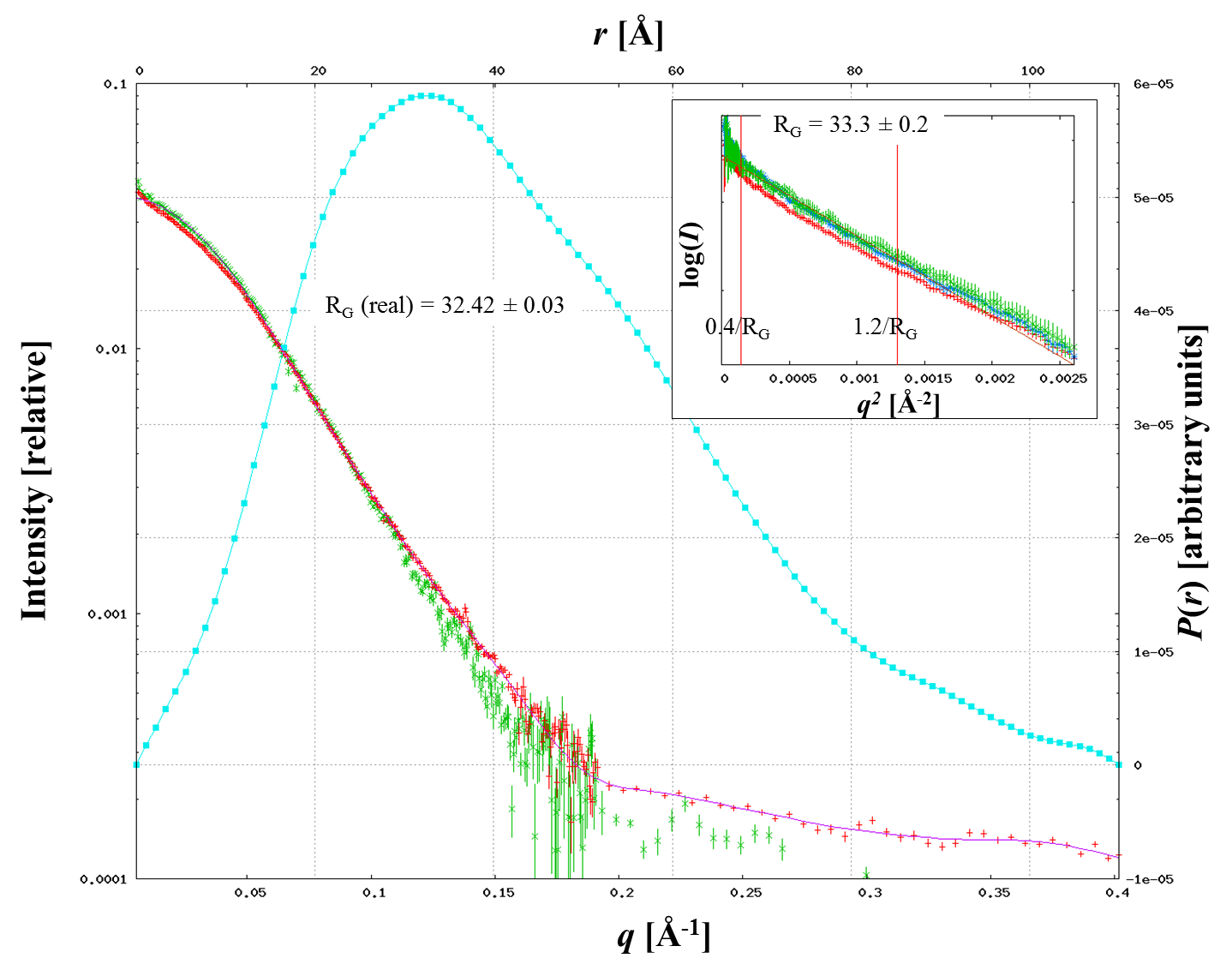

Supplement: Figure S1 — Scattering curves I(q), pairwise function P(r) and Guinier plots (inset) for S. elongatus KaiA. Scattering curves: red with error bars (from GNOM) = high concentration, 2.1 mg/mL; green with error bars (from GNOM) = low concentration, 0.85 mg/mL; magenta line = FT of GNOM scan P(r). The cyan curve corresponds to P(r) from GNOM (Svergiun, 1992). Inset: red with error bars = high conc., 2.1 mg/mL; blue with error bars = medium conc., 1.5 mg/mL; green with error bars = low conc., 0.85 mg/mL; brown line = Guinier fit of data between the red bars (0.4/RG to 1.2/RG). (TIF) [file pone.0023697.s001.tif]

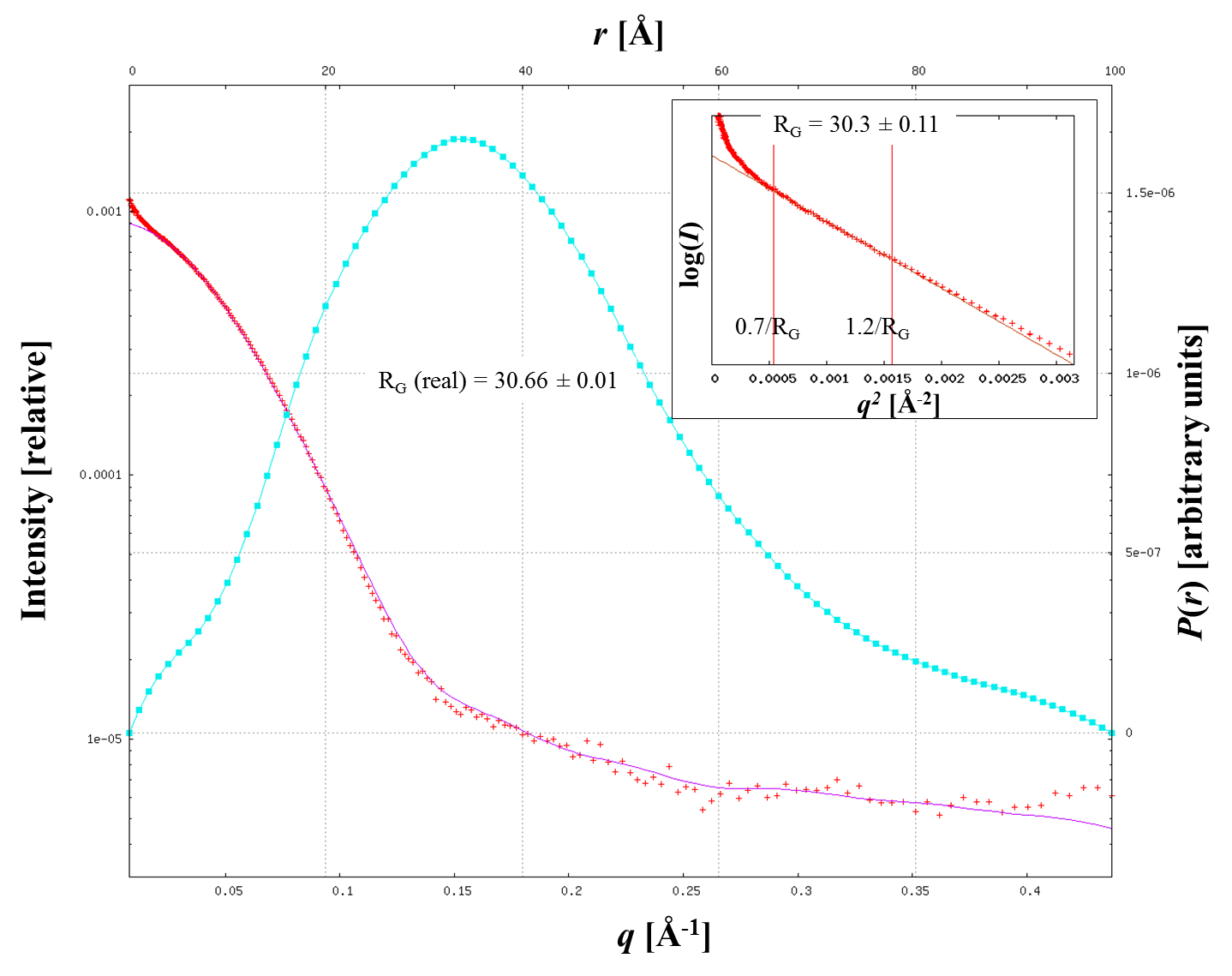

Supplement: Figure S2 — Scattering curve I(q), pairwise function P(r) and Guinier plot (inset) for S. elongatus KaiB. Scattering curve: red with error bars, concentration 1 mg/mL; magenta line = FT of GNOM scan P(r). The cyan curve corresponds to P(r) from GNOM (Svergun, 1992). Inset: red with error bars = conc. 1 mg/mL; brown line = Guinier fit of data between the red bars (0.7/RG to 1.2/RG). (TIF) [file pone.0023697.s002.tif]

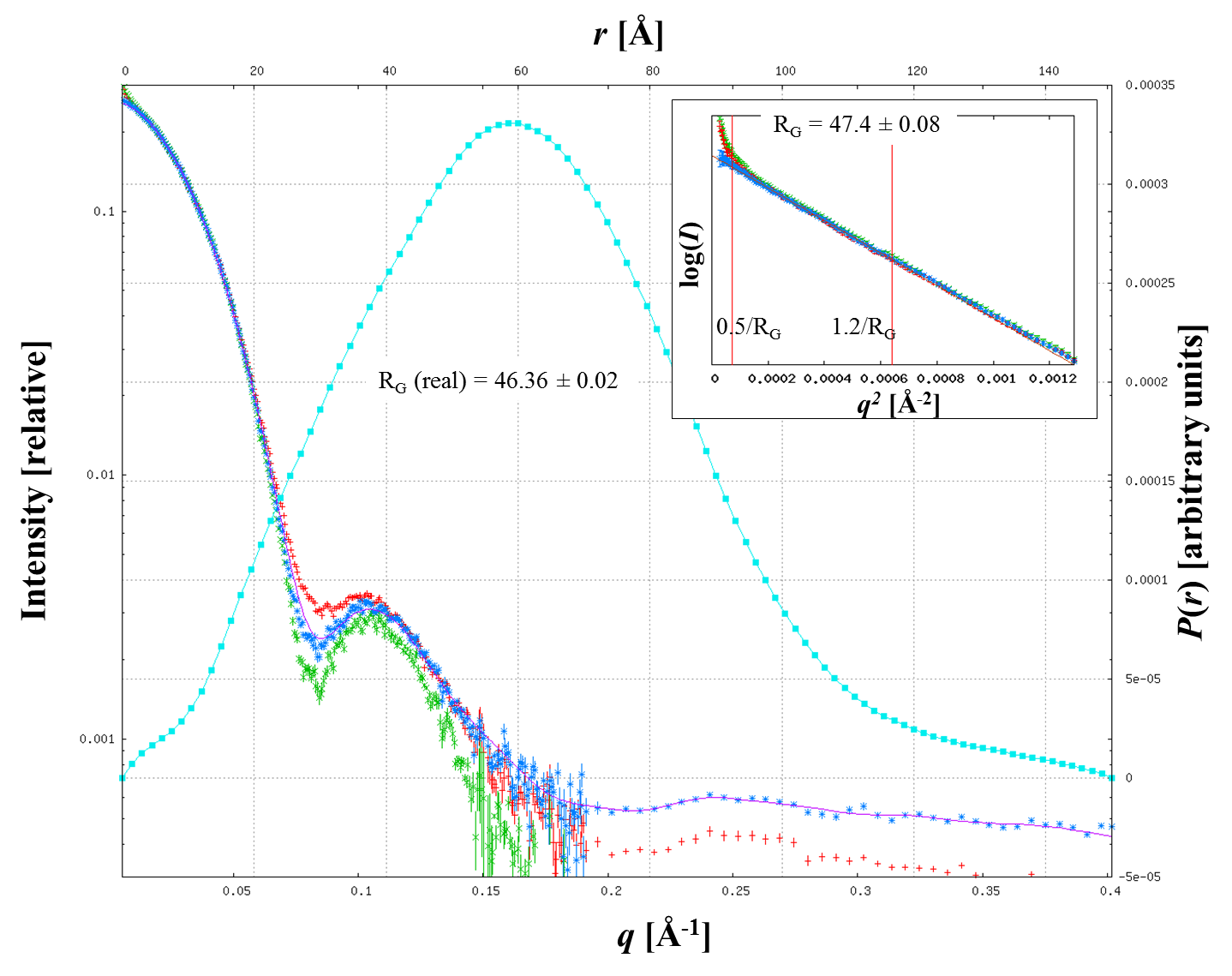

Supplement: Figure S3 — Scattering curves I(q), pairwise function P(r) and Guinier plots (inset) for S. elongatus KaiC. Scattering curves: red with error bars (from GNOM) = high concentration, 0.95 mg/mL; blue with error bars (from GNOM) = medium concentration, 0.80 mg/mL; green with error bars (from GNOM) = low concentration, 0.66 mg/mL; magenta line = FT of GNOM scan P(r). The cyan curve corresponds to P(r) based on the medium concentration from GNOM (Svergun, 1992). Inset: red with error bars = high conc., 0.95 mg/mL; blue with error bars = medium conc., 0.80 mg/mL; green with error bars = low conc., 0.66 mg/mL; brown line = Guinier fit of data between the red bars (0.5/RG to 1.2/RG). (TIF) [file pone.0023697.s003.tif]

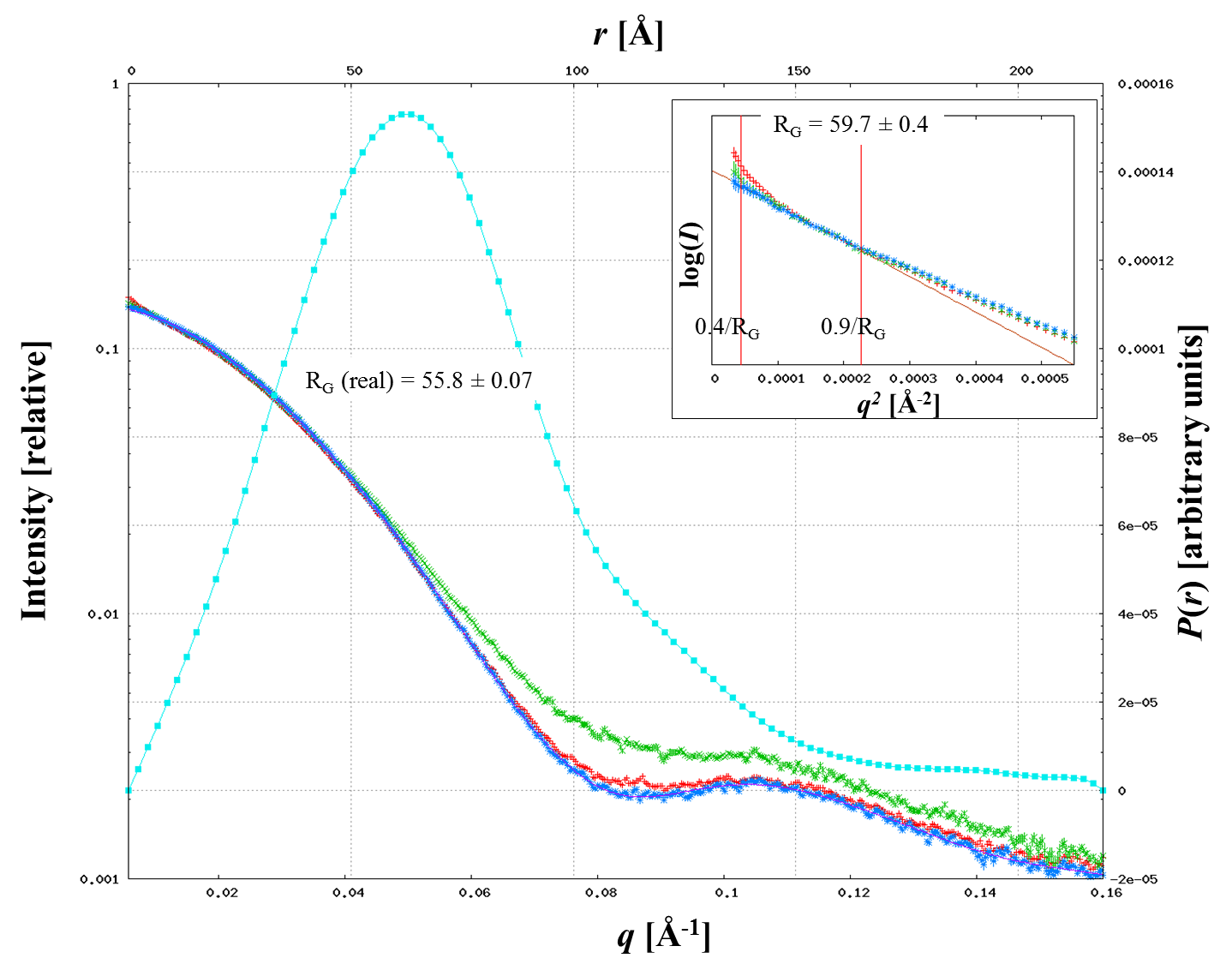

Supplement: Figure S4 — Scattering curves I(q), pairwise function P(r) and Guinier plots (inset) for the S. elongatus KaiAC complex (KaiC-aa mutant). Scattering curves: red with error bars = high concentration, 2.1 mg/mL; blue with error bars = medium concentration, 1.6 mg/mL; green with error bars = low concentration, 1.1 mg/mL; magenta line = FT of GNOM med conc. P(r). The cyan curve corresponds to P(r) from GNOM (Svergun, 1992). Inset: red with error bars = high conc. (2.1 mg/mL); blue with error bars = medium conc. (1.6 mg/mL); green with error bars = low conc., 1.1 mg/mL; brown line = Guinier fit of data between the red bars (0.4/RG to 0.9/RG). (TIF) [file pone.0023697.s004.tif]

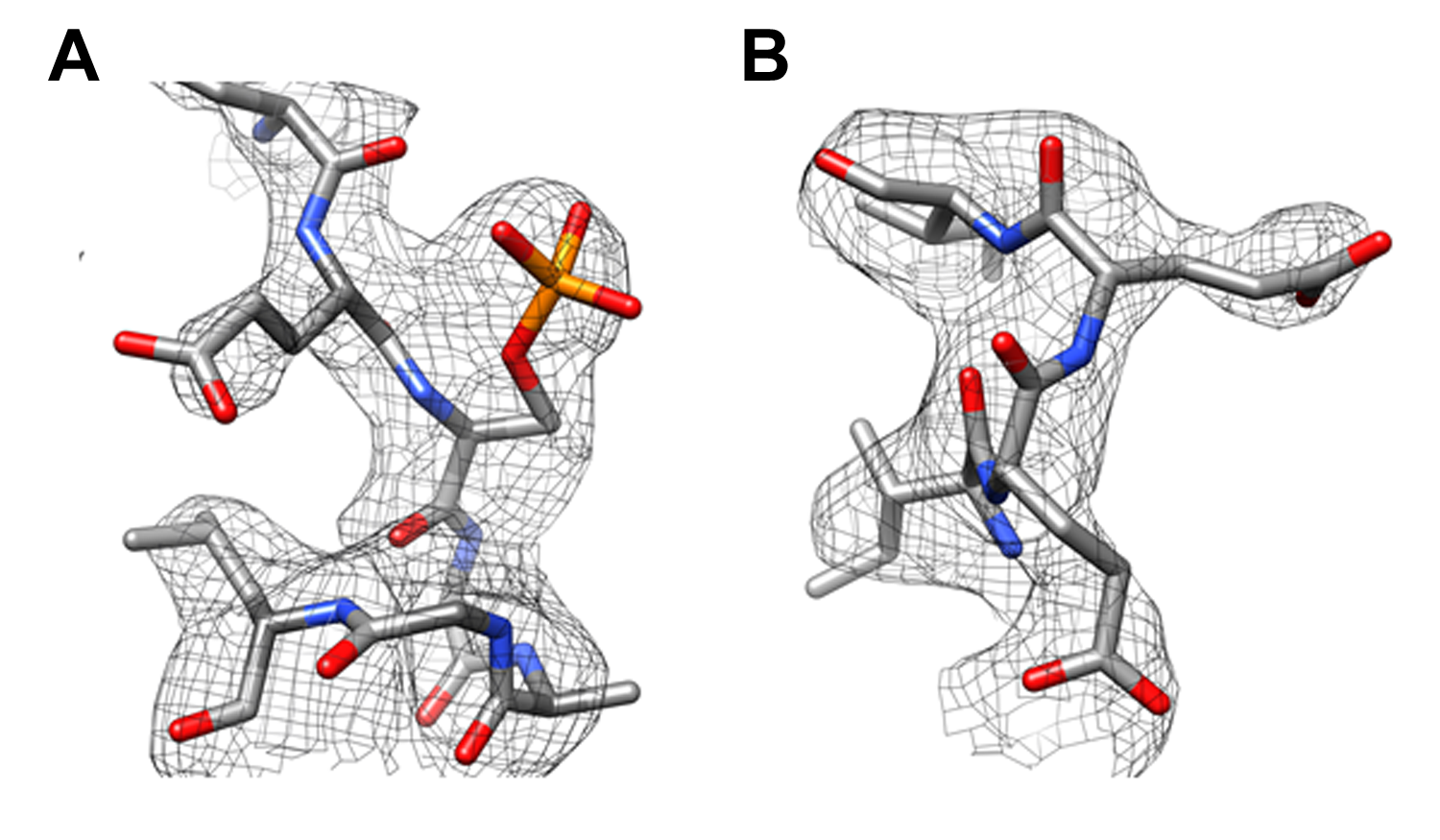

Supplement: Figure S5 — Quality of the final crystallographic model for the KaiC-ee mutant. Fourier sum (2Fo-Fc) density contoured at the 1σ level around (A) residue pS320 in the F subunit (phospho-serine; upper right), and (B) around E431 (lower right) and E432 (upper right) in the A subunit. (TIF) [file pone.0023697.s005.tif]

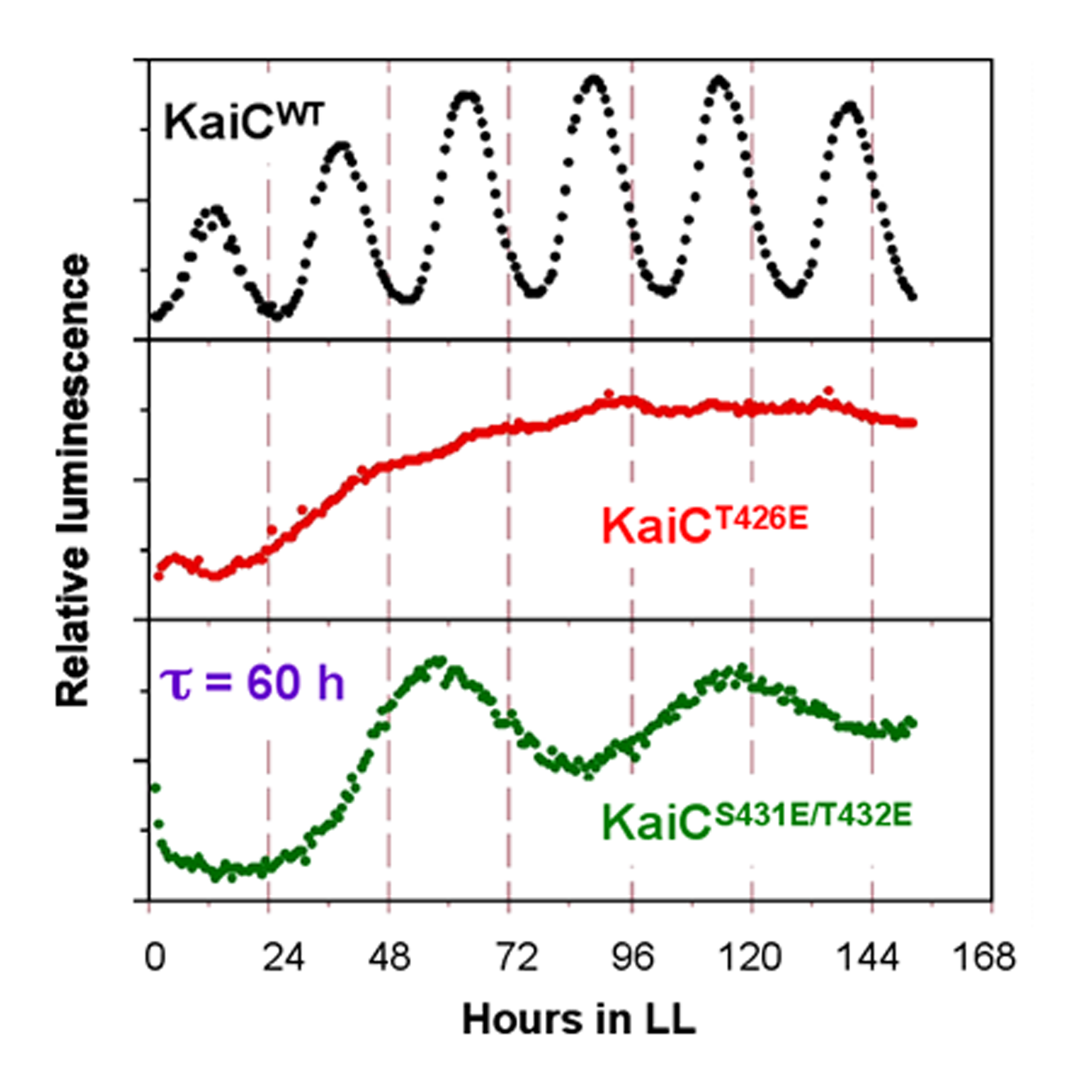

Supplement: Figure S6 — Effect of Glu substitution of KaiC P-sites on the PkaiBC-driven rhythm. Following a dark synchronization, luminescence was measured in reporter strains expressing either wt-KaiC (KaiCWT) or the KaiC-ee and T426E mutants (KaiCS431E/T432E and KaiCT426E, respectively). (TIF) [file pone.0023697.s006.tif]

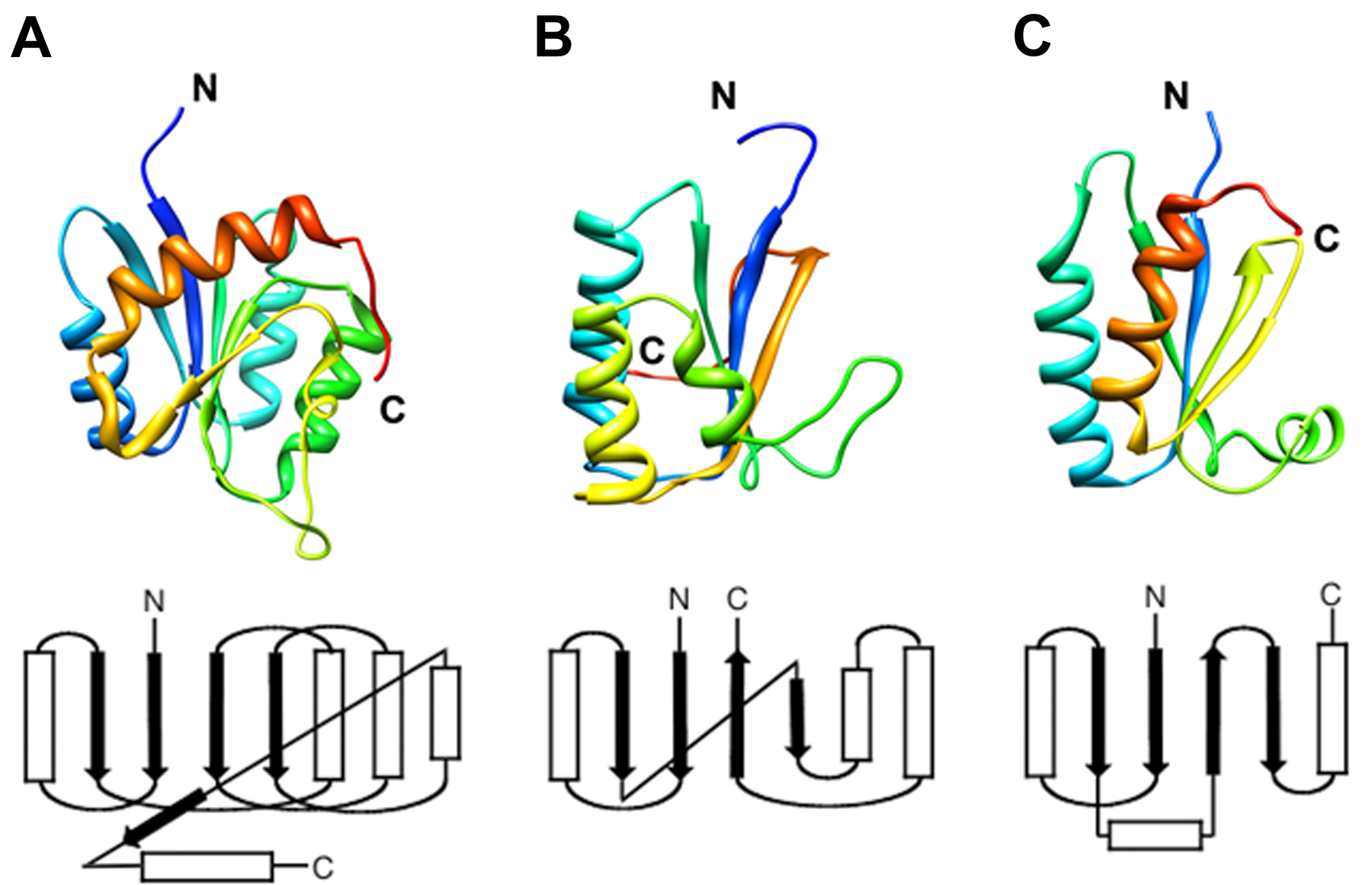

Supplement: Figure S7 — Comparisons between the three-dimensional folds (N-terminus blue to C-terminus red) of (A) N-KaiA [S. elongatus; PDB 1R8J; X-ray (Ye et al., 2004)], (B) KaiB [T. elongatus; PDB ID 2QKE; X-ray (Pattanayek et al., 2008)], and (C) N-SasA (S. elongatus; PDB ID 14TY; NMR (Vakonakis et al., 2004)]. (TIF) [file pone.0023697.s007.tif]

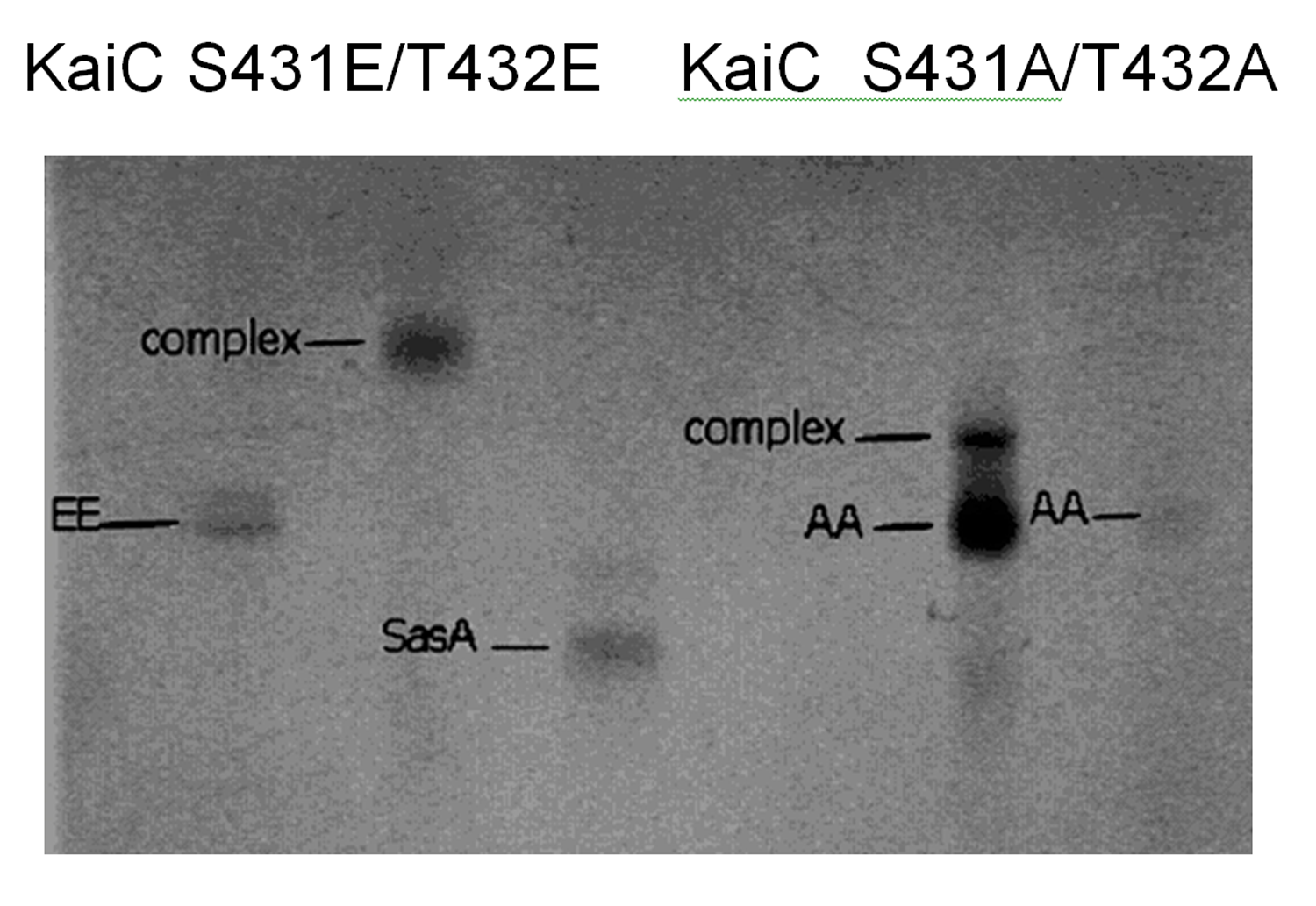

Supplement: Figure S8 — Native PAGE assays for complex formation between either KaiC-ee (left) or KaiC-aa (right) and full-length SasA. (TIF) [file pone.0023697.s008.tif]

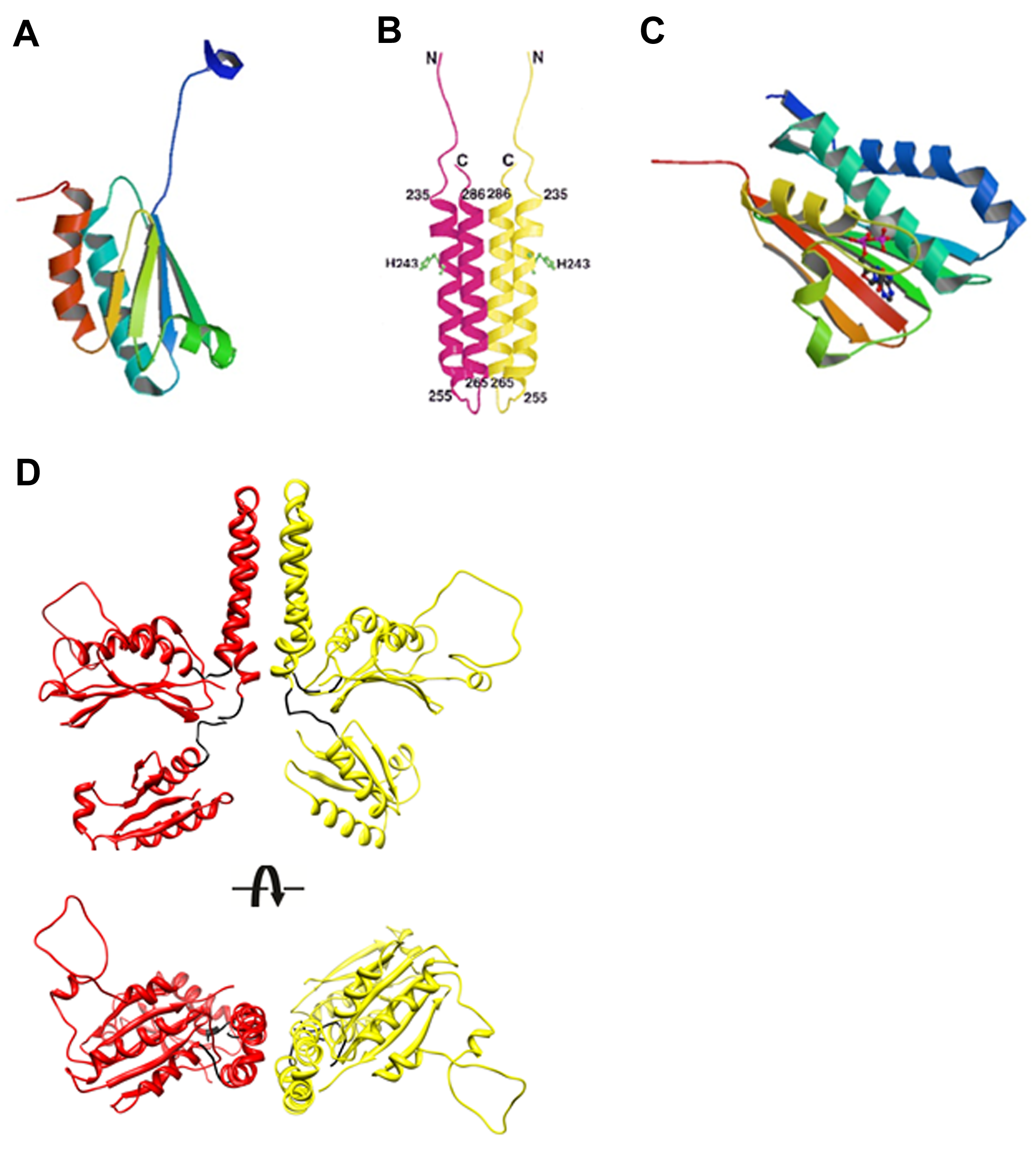

Supplement: Figure S9 — Three-dimensional structures and models of His kinase domains. (A) N-terminal sensory domain of S. elongatus SasA [PDB ID 1T4Y; NMR (Vakonakis et al. 2004)], (B) dimerization domain harboring the His phosphorylation site of E. coli EnvZ [PDB ID 1JOY; NMR (Tomomori et al., 1999)], and (C) the catalytic domain of E. coli EnvZ with bound ADP[PDB ID 1BXD; NMR (Tanaka et al., 1998)]. (D) Model of the full-length SasA dimer, viewed perpendicularly to the molecular dyad, and rotated by 90° and viewed along the molecular dyad. Linker regions are highlighted in black. (TIF) [file pone.0023697.s009.tif]

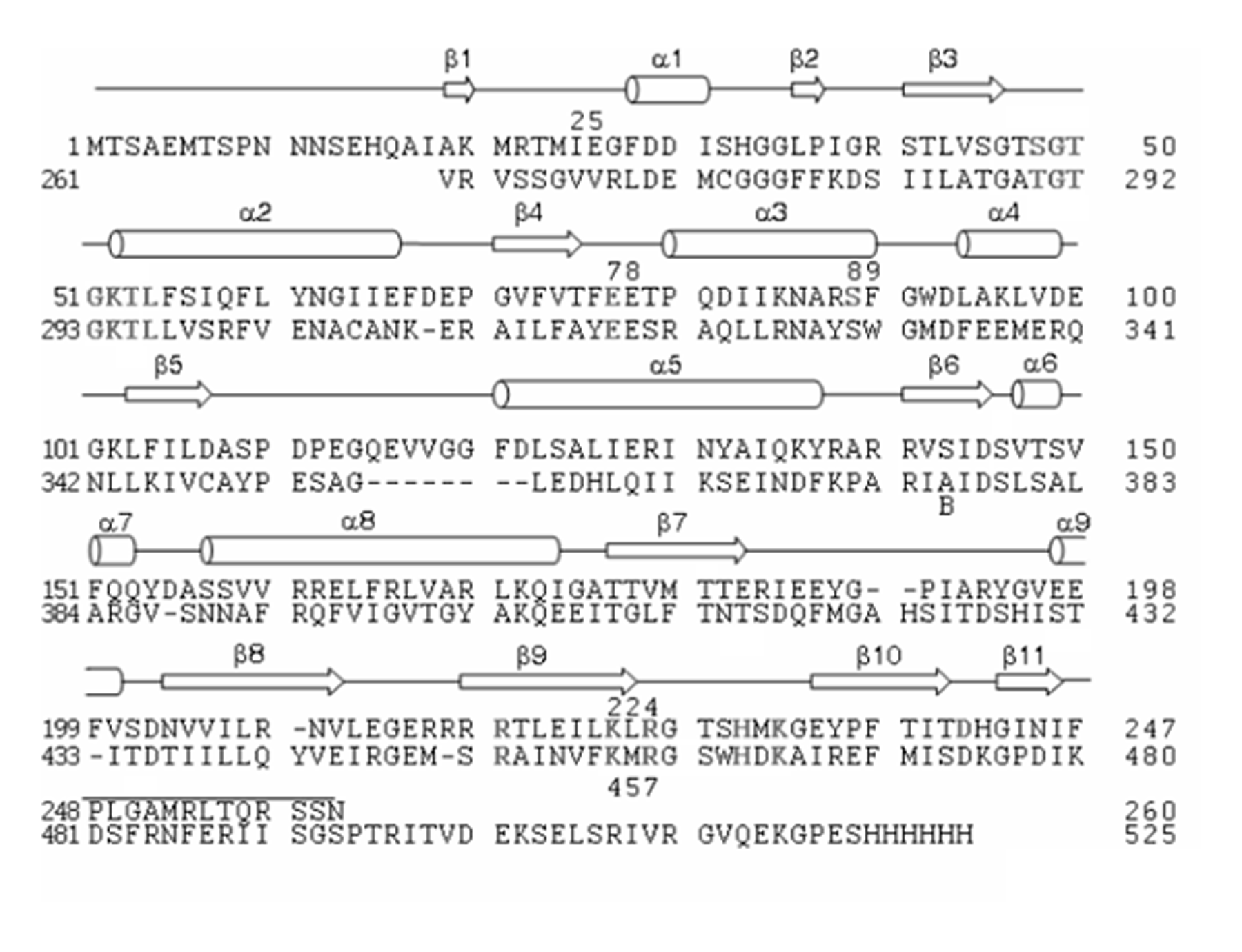

Supplement: Figure S10 — Sequence alignment and secondary structures of S. elongatus KaiCI (upper line) and KaiCII (lower line). (TIF) [file pone.0023697.s010.tif]
