# Supplementary material for: Combined SAXS/EM Based Models of the S. elongatus Post-Translational Circadian Oscillator and its Interactions with the Output His-Kinase SasA
Source: PLoS One. 2011 Aug 24;6(8):e23697. doi: 10.1371/journal.pone.0023697 (PMC3161067; doi:10.1371/journal.pone.0023697)
Supplement: Table S1 — Selected crystal data, X-ray data collection and refinement parameters for the crystal structure of S. elongatus KaiC-eea. (DOCX) [file pone.0023697.s011.docx]

**Table S1.** Selected crystal data, X-ray data collection and refinement parameters for the crystal structure of *S. elongatus* KaiC-ee*^a^*.

| **Parameter** | **KaiC-ee** |
| --- | --- |
| Space group | *P*2_1_2_1_2_1_ |
| Unit cell |  |
| *a* [Å] | 132.67 |
| *b* [Å] | 135.49 |
| *c* [Å] | 204.60 |
| Resolution [Å] | 3.00 |
| Completeness [%] | 99.4 |
| Outer shell* [%] | 96.3 |
| *Resolution range [Å] | 3.06-3.00 |
| I/σ(I) (outer shell) | 3.3 |
| R-merge [%]  Outer shell [%] | 7.5  38.1 |
| R-work [%] | 24.2 |
| R-free [%] | 28.8 |
| Reflections used for R-free [%] | 8.7 |
| Number of atoms | 23,819 |
| Number of ATP molecules | 12 |
| Number of solvent molecules | 95 |
| R.m.s.d bonds [Å] | 0.01 |
| R.m.s.d angles [°] | 1.6 |

^a^ Diffraction data were collected on the 21-ID-F beamline at the APS (Argonne National Laboratory, Argonne, IL), using a MAR300 CCD detector. The data collection temperature was 110K.
