# Supplementary material for: Combined SAXS/EM Based Models of the S. elongatus Post-Translational Circadian Oscillator and its Interactions with the Output His-Kinase SasA
Source: PLoS One. 2011 Aug 24;6(8):e23697. doi: 10.1371/journal.pone.0023697 (PMC3161067; doi:10.1371/journal.pone.0023697)
Supplement: References S1 — Supplementary References. (DOCX) [file pone.0023697.s012.docx]

**
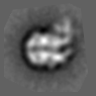

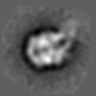

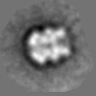

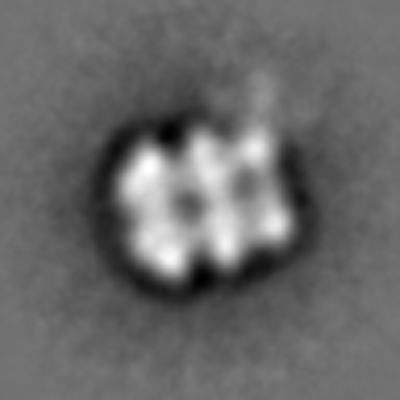

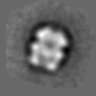

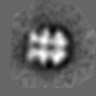
Supporting Information**

*All are tri-layered, suggesting binding to the same side of the KaiC hexamer*

*Mixtures of KaiB, KaiC, and SasA*

Combined SAXS/EM Based Models of the *S. elongatus* Post-Translational Circadian Oscillator and its Interactions with the Output His-Kinase SasA

Rekha Pattanayek^1^, Dewight R. Williams^2^, Gian Rossi^1^, Steven Weigand^3^, Tetsuya Mori^4^, Carl H. Johnson^2,4^, Phoebe L. Stewart^2^, Martin Egli^1,*^

**^1^** Department of Biochemistry, School of Medicine, Vanderbilt University, Nashville, Tennessee 37232, United States of America, **^2^** Department of Molecular Physiology and Biophysics, School of Medicine, Vanderbilt University, Nashville, Tennessee 37232, United States of America, **^3^** DND-CAT Synchrotron Research Center, Northwestern University, Advanced Photon Source, Argonne National Laboratory, Argonne, Illinois 60439, United States of America, **^4^** Department of Biological Sciences, Vanderbilt University, Nashville, Tennessee 37235, United States of America

**Supplementary References**

Pattanayek R, Williams DR, Pattanayek S, Mori T, Johnson CH, Stewart PL, Egli M (2008) Structural model of the circadian clock KaiB-KaiC complex and mechanism for modulation of KaiC phosphorylation. *EMBO J* **27:** 1767-1778

Svergun DI (1992) Determination of the regularization parameter in indirect-transform methods using perceptual criteria. *J Appl Cryst* **25:** 495-503

# [Tanaka T](http://www.ncbi.nlm.nih.gov/pubmed?term=%22Tanaka%20T%22%5BAuthor%5D), [Saha SK](http://www.ncbi.nlm.nih.gov/pubmed?term=%22Saha%20SK%22%5BAuthor%5D), [Tomomori C](http://www.ncbi.nlm.nih.gov/pubmed?term=%22Tomomori%20C%22%5BAuthor%5D), [Ishima R](http://www.ncbi.nlm.nih.gov/pubmed?term=%22Ishima%20R%22%5BAuthor%5D), [Liu D](http://www.ncbi.nlm.nih.gov/pubmed?term=%22Liu%20D%22%5BAuthor%5D), [Tong KI](http://www.ncbi.nlm.nih.gov/pubmed?term=%22Tong%20KI%22%5BAuthor%5D), [Park H](http://www.ncbi.nlm.nih.gov/pubmed?term=%22Park%20H%22%5BAuthor%5D), [Dutta R](http://www.ncbi.nlm.nih.gov/pubmed?term=%22Dutta%20R%22%5BAuthor%5D), [Qin L](http://www.ncbi.nlm.nih.gov/pubmed?term=%22Qin%20L%22%5BAuthor%5D), [Swindells MB](http://www.ncbi.nlm.nih.gov/pubmed?term=%22Swindells%20MB%22%5BAuthor%5D), [Yamazaki T](http://www.ncbi.nlm.nih.gov/pubmed?term=%22Yamazaki%20T%22%5BAuthor%5D), [Ono AM](http://www.ncbi.nlm.nih.gov/pubmed?term=%22Ono%20AM%22%5BAuthor%5D), [Kainosho M](http://www.ncbi.nlm.nih.gov/pubmed?term=%22Kainosho%20M%22%5BAuthor%5D), [Inouye M](http://www.ncbi.nlm.nih.gov/pubmed?term=%22Inouye%20M%22%5BAuthor%5D), [Ikura M](http://www.ncbi.nlm.nih.gov/pubmed?term=%22Ikura%20M%22%5BAuthor%5D) (1998) NMR structure of the histidine kinase domain of the E. coli osmosensor EnvZ. *Nature* 396: 88-92

1. Tomomori C, Tanaka T, Dutta R, Park H, Saha SK, Zhu Y, Ishima R, Liu D, Tong, KI, Kurokawa H, Qian H, Inouye M, Ikura M. (1999) Solution structure of the homodimeric core domain of Escherichia coli histidine kinase EnvZ. *Nat Struct Biol* **6:** 729-734

Vakonakis I, Klewer DA, Williams SB, Golden SS, LiWang AC (2004) Structure of the N-terminal domain of the circadian clock-associated histidine kinase SasA. *J Mol Biol* **342:** 9-17

Ye S, Vakonakis I, Ioerger TR, LiWang AC, Sacchettini JC (2004) Crystal structure of circadian clock protein KaiA from Synechococcus elongatus. *J Biol Chem* **279:** 20511-20518
